# Crystal structure of (6,9-diacetyl-5,10,15,20-tetra­phenyl­secochlorinato)nickel(II)

**DOI:** 10.1107/S2056989024004717

**Published:** 2024-05-24

**Authors:** Meenakshi Sharma, Christian Brückner, Matthias Zeller

**Affiliations:** a Department of Chemistry, University of Connecticut, Unit 3060, Storrs, CT, 06269-3060, USA; b Department of Chemistry, Purdue University, 560 Oval Drive, West Lafayette, IN, 47907-2084, USA; University of Massachusetts Dartmouth, USA

**Keywords:** crystal structure, porphyrinoids, *meso*-phenyl­secochlorins, pyrrole-modified porphyrins

## Abstract

Title compound **1Ni**, [Ni(C_46_H_32_N_4_O_2_)], a secochlorin nickel complex, was prepared by diol cleavage of a precursor *trans*-di­hydroxy­dimethyl­chlorin. Two crystallographically independent mol­ecules in the structure are related by pseudo-A lattice centering, with mol­ecules differing mainly by a rotation of one of the acetyls and an adjacent phenyl groups. The two mol­ecules have virtually identical conformations characterized by noticeable in-plane deformation in the A_1g_ mode and a prominent out-of-plane deformation in the B_1u_ (ruffling) mode. Directional inter­actions between mol­ecules are scarce, limited to just a few C—H⋯O contacts, and inter­molecular inter­actions are mostly dispersive in nature.

## Abstract 2

The structure of the title secochlorin nickel complex features two crystallographically independent mol­ecules related by pseudo-A lattice centering. Both feature a noticeable in-plane deformation in the A_1g_ mode and prominent out-of-plane deformation in the B_1u_ (ruffling) mode.

## 1. Chemical context

Chlorins are 7,8-di­hydro­porphyrins, *i.e.*, porphyrins reduced at one of the pyrrolic β,β′-double bonds (Borbas, 2016 ▸; Taniguchi & Lindsey, 2017 ▸; Lindsey, 2015 ▸). The chloro­phylls, nature’s light harvesting pigments, are magnesium complexes of chlorins (Taniguchi & Lindsey, 2017 ▸). In large part because of the role of this member of the ‘pigments of life’ (Battersby, 2000 ▸) in photosynthesis, chlorins are a broadly investigated compound class.

Secochlorins are compounds in which a pyrrolic β,β′-double bond was cleaved (Brückner *et al.*, 2014 ▸; Thuita & Brückner, 2022 ▸). These compounds have no known precedent among the natural porphyrinic pigments or their degradation products (Kräutler, 2014 ▸; Wojaczyński, 2014 ▸). The handful of examples of secochlorins prepared to date were made by oxidative modifications of functionalized porphyrin or chlorin β,β′-bonds (Brückner *et al.*, 2014 ▸; Thuita & Brückner, 2022 ▸). The first secochlorin was discovered fortuitously (Chang *et al.*, 1992 ▸). Since then, a number of rational oxidative β,β′-bond cleavage reaction sequences have been developed to prepare (metallo)secochlorins of the octa­alkyl- (Adams *et al.*, 1997 ▸; Sessler *et al.*, 2001 ▸; Ryppa *et al.*, 2009 ▸) and the *meso*-tetra­aryl­porphyrin series (Brückner *et al.*, 1998 ▸, 1999 ▸, 2005 ▸; McCarthy *et al.*, 2004 ▸; Akhigbe *et al.*, 2009 ▸; Sharma *et al.*, 2016 ▸; Lo *et al.*, 2012 ▸). Following a β,β′-bond-opening, subsequent intra­molecular reactions of the secochlorins with the adjacent *meso*-aryl- or β-alkyl groups are not infrequent (Adams *et al.*, 1997 ▸; McCarthy *et al.*, 2004 ▸; Ryppa *et al.*, 2009 ▸; Banerjee *et al.*, 2012 ▸; Zhu *et al.*, 2022 ▸).

**Figure 1:**
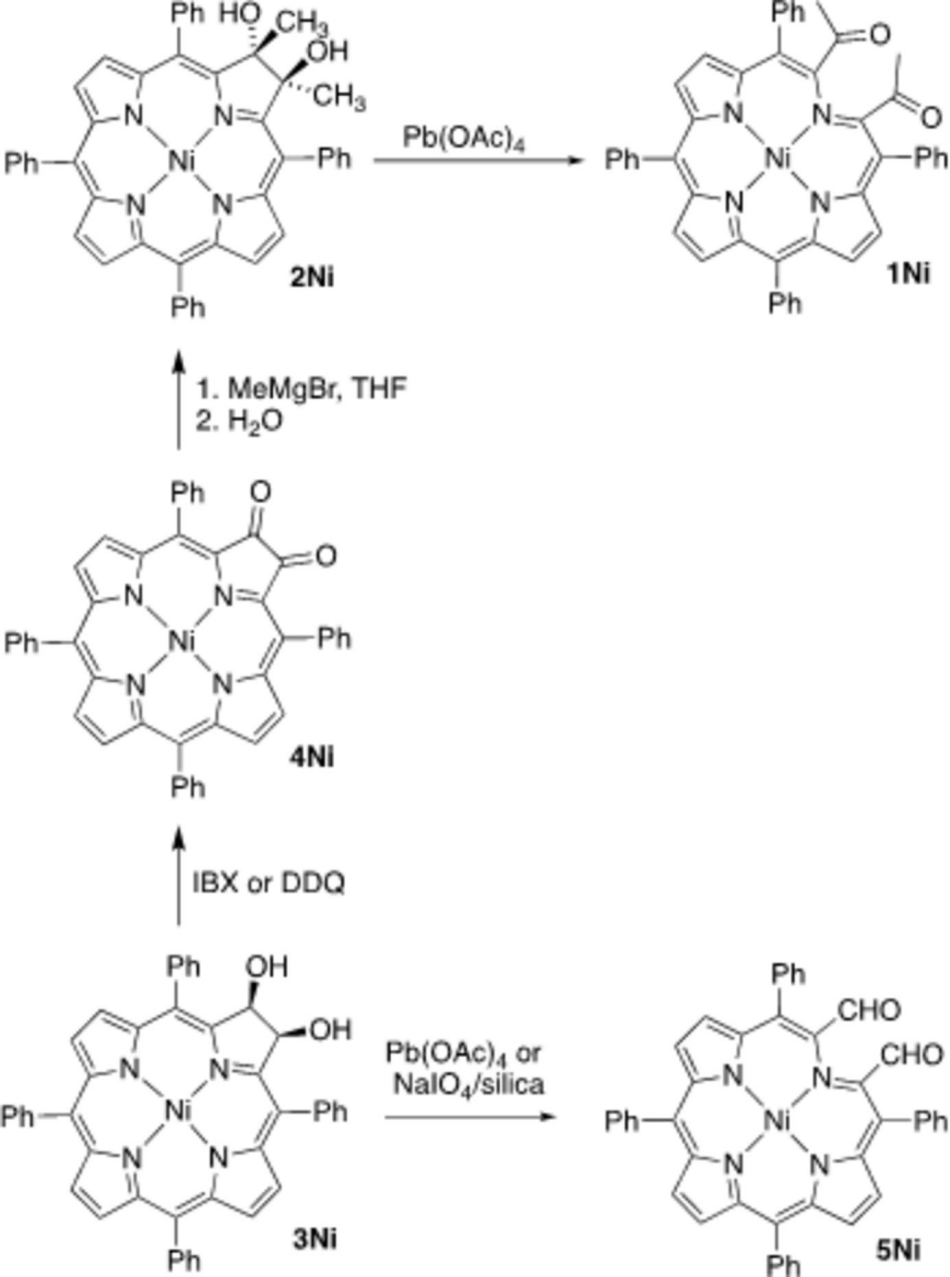
Synthetic pathway to secochlorin nickel(II) complexes **1Ni** and **5Ni** by oxidative diol cleavage of a precursor diol.

The title compound **1Ni**, a secochlorin nickel complex, was prepared by diol cleavage of *trans-*di­hydroxy­chlorin **2Ni**, itself made in a multi-step process from *cis*-diol chlorin **3Ni** (Fig. 1 ▸). It has been used in the preparation of a number of porphyrinoids containing non-pyrrolic heterocycles (Banerjee *et al.*, 2012 ▸; Sharma *et al.*, 2016 ▸). 
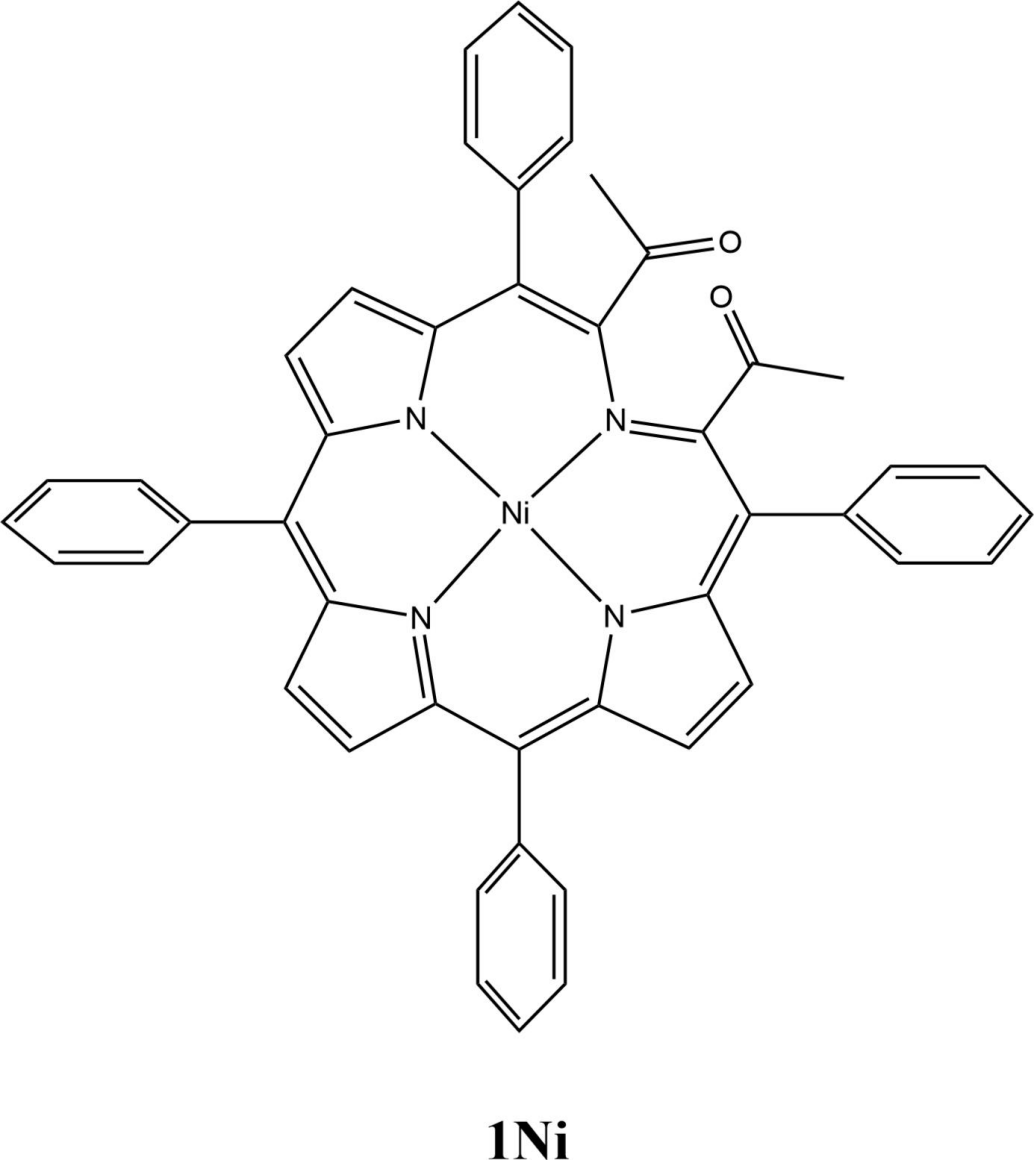


Chlorin diol **3Ni** could also be cleaved, generating bis­aldehyde **5Ni** (Brückner *et al.*, 1998 ▸, 1999 ▸, 2005 ▸). A comparison of the crystal structures of the di­hydroxy­chlorins **2Ni**/**3Ni** and the corresponding secochlorins **1Ni**/**5Ni** provides further insight into the conformational changes induced by breaking the structural integrity of the porphyrin framework (Brückner *et al.*, 2005 ▸) and the influence of β-alkyl­ation.

## 2. Structural commentary

**Figure 2:**
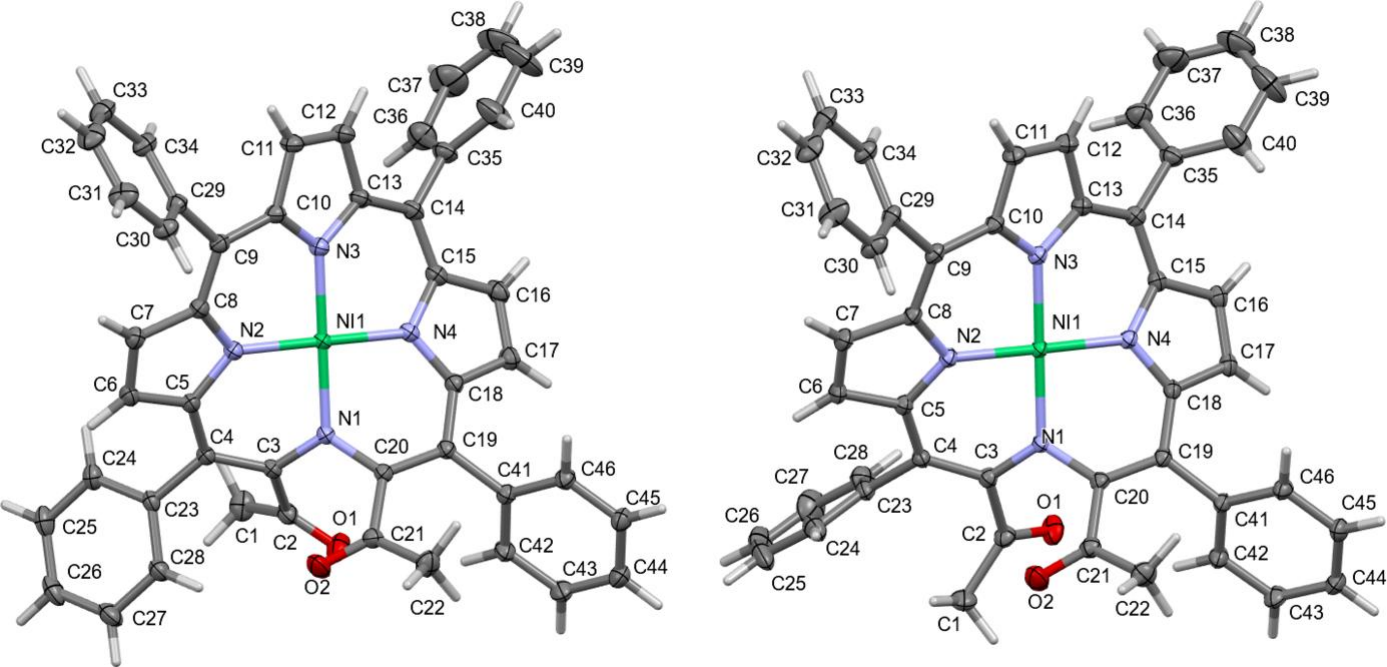
Displacement ellipsoid view of the two mol­ecules of the structure of compound **1Ni** with atom-labeling scheme (50% probability ellipsoids). Left: mol­ecule **1Ni-1**. Right: Mol­ecule **1Ni-2**.

**1Ni** crystallized in a centrosymmetric triclinic setting in space group *P* 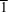 , with two crystallographically independent mol­ecules (*Z*′ = 2) (Fig. 2 ▸). The structure of **1Ni** confirms its rare secochlorin bis­ketone connectivity that was previously derived spectroscopically and by means of subsequent reactions (Banerjee *et al.*, 2012 ▸; Sharma *et al.*, 2016 ▸). Bond lengths and angles are in the expected ranges. Noteworthy, however, are the C—N bonds around the cleavage site at N1, which show a clear asymmetry present in both mol­ecules. The lengths for N1—C3 are 1.3687 (15) and 1.3661 (15) Å in the two mol­ecules **1Ni-1** and **1Ni-2**, while the two N1—C20 bonds are substanti­ally longer at 1.3879 (15) and 1.3997 (15) Å. For most of the other bonds of the secochlorin skeleton no such clear differentiation is observed.

**Figure 3:**
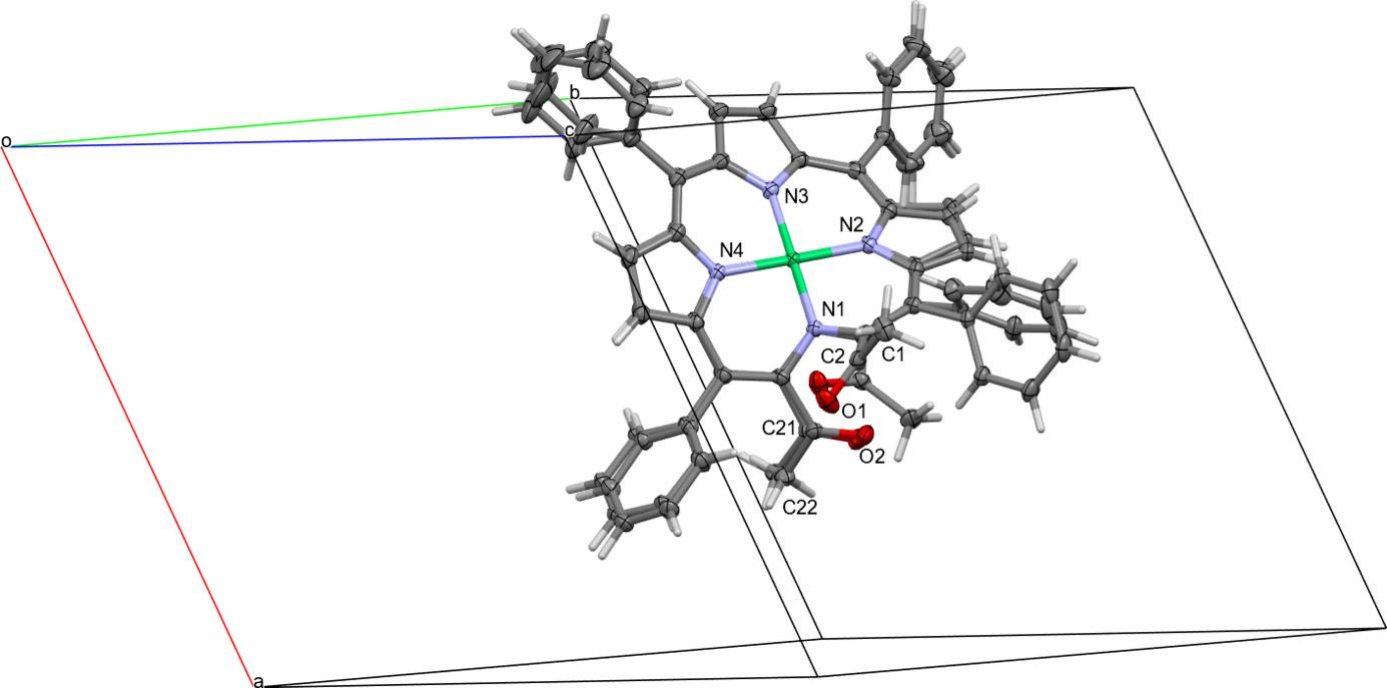
View down [01 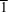 ] (slightly offset) showing the pseudo-A lattice centering relating the two mol­ecules with each other (front: mol­ecule **1Ni-1**; back: mol­ecule **1Ni-2**). For clarity only selected labels for mol­ecule 1 are shown.

The two crystallographically independent mol­ecules of the secochlorinato nickel complex **1Ni** are related by pseudo-A lattice centering (Fig. 3 ▸). Exact translational symmetry is broken by different torsion angles for one of the acetyl groups (O1, C1, C2). In mol­ecule 1, the N—C—C—O torsion angle between the non-pyrrolic N atom N1 and keto-oxygen O1 is 39.90 (19)°; in mol­ecule 2 the same torsion angle is −37.75 (17)°. The same torsion angles for the methyl C atom C1 instead of O1 are 143.45 (13)° (mol­ecule 1) and −130.33 (13)° (mol­ecule 2). The acetyl groups of the two mol­ecules are thus rotated with respect to each other, by *ca* 78 and 86° for keto O and methyl C atoms, respectively. The different values for C and O atoms are caused by a slight non-planarity of the acetyl group of mol­ecule 1, with C1 being offset from the plane of the C3, C2 and O1 by 0.230 (5) Å. This rotation results in a significantly different O1⋯O2 distance in the two mol­ecules: 2.7997 (17) Å in mol­ecule 1, and 3.2286 (16) Å in mol­ecule 2. It also places the methyl groups in the two mol­ecules on opposite sides of their neighboring phenyl rings of C23 to C28, which in turn induces different orientations of this ring between the two mol­ecules [C3—C4—C23—C24 = −130.42 (14) and 115.13 (15)° for mol­ecules 1 and 2, respectively]. The different orientation of the phenyl ring prevents an intra­molecular C—H⋯O hydrogen bond between C28 and O2 that is present in mol­ecule 1 to be formed in mol­ecule 2. A second C—H⋯O hydrogen bond (between C42 and O1) is present for both mol­ecules (see Table 1 ▸ for numerical details). The phenyl group on the opposite end of the mol­ecule (C35–C40) is also slightly modulated, but much less so than the acetyl group and C23–C28 (torsion angles only differ by *ca* 29°). The remaining atoms of the two mol­ecules are related by close to perfect translational symmetry, and the conformations of their secochlorin cores are thus qualitatively and qu­anti­tatively very similar to each other.

**Figure 4:**
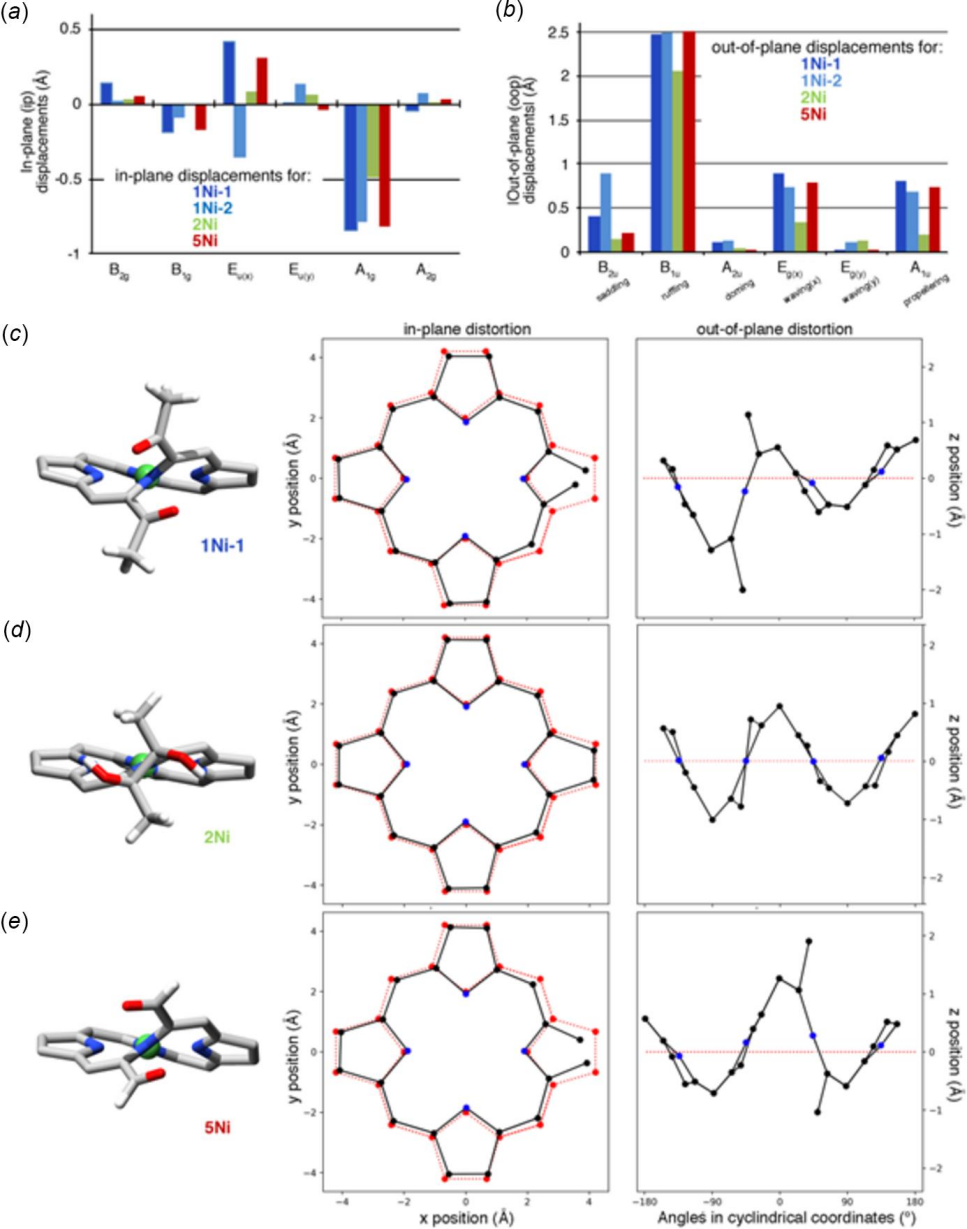
Bar diagrams of the in-plane (*a*) and out-of-plane (*b*) macrocycles NSD deformation analyses of the compounds **1Ni-1**, **1Ni-2**, **2Ni**, and **5Ni**; analyses by the porphyrin NSD online tool (Kingsbury & Senge, 2021 ▸), based on the method of Shelnutt (Jentzen *et al.*, 1997 ▸). Stick representations of the X-ray single-crystal structures are shown below (*c* through *e*); for **1Ni**, only **1Ni-1** is shown; all H atoms attached to *sp* ^3^-hybridized carbon atoms, all *meso*-aryl groups, as well as all disorder and solvents removed for clarity. Next to the stick structures are the in-plane skeletal plots of the porphyrin core of the compounds indicated (black trace), compared to that of a benchmark planar porphyrin [*meso*-tetra­phenyl­porphyrinato]copper(II) (red trace), as well as their out-of-plane skeletal plots.

Importantly, the structure determination of **1Ni** also allows for a detailed analysis of the conformation of the macrocycle. For both mol­ecules **1Ni-1** and **1Ni-2** the conformations of the chromophores deviate greatly from planarity. On account of the small central nickel(II) ion inducing strain into the macrocycle, nickel(II) porphyrin and chlorin complexes tend to be non-planar, adopting commonly a ruffled conformation (Kingsbury & Senge, 2021 ▸). This is because the ruffled deformation mode effectively shortens the Ni—N bond lengths, without distorting the near-ideal planar coordination geometry of the four nitro­gen donors around the metal. This is also the case here, and the N—Ni bond lengths vary only within a small margin, with values between 1.8845 (10) and 1.9128 (10) Å, and the coordination of the nickel atoms is close to perfectly square planar. To compare **1Ni** to other related compounds and obtain a qualitative and qu­anti­tative analysis of its conformation, we performed a normal-coordinate structural decomposition (NSD) analysis of the macrocycle conformation of the two mol­ecules of **1Ni-1** and **1Ni-2**, of the starting diol nickel complex **2Ni** and of its aldehyde analogue **5Ni** (Fig. 4 ▸) (Jentzen *et al.*, 1997 ▸; Kingsbury & Senge, 2021 ▸).

Both mol­ecules **1Ni-1** and **1Ni-2** show in-plane compressed conformations, with the most prominent in-plane deformation in the A_1g_ mode. This is combined with drastically ruffled out-of-plane deformations with significant waving and propellering contributions. These deformation modes are typical for nickel porphyrins and chlorins (Kingsbury & Senge, 2021 ▸) and their large extent was previously observed in secochlorins (Brückner *et al.*, 2005 ▸). The macrocycle conformation aligns the two ketone functionalities to be arranged anti­parallel to each other. Qualitatively and qu­anti­tatively, the conformation of bis­ketone **1Ni** is very similar to that of bis­aldehyde **5Ni**, showing that the additional alkyl substituents in **1Ni** do not have any significant steric influence. While parent chlorin diol complex **2Ni** is also ruffled, it is much less so, with much smaller waving and propellering deformations as well. Accordingly, the average N—Ni bond length in **2Ni** is longer (1.908 Å) compared to the corresponding bond lengths found in **1Ni** (1.901 Å) or **5Ni** (1.893 Å). The changes observed upon ring-opening align with what was previously observed (Brückner *et al.*, 2005 ▸).

## 3. Supra­molecular features

**Figure 5:**
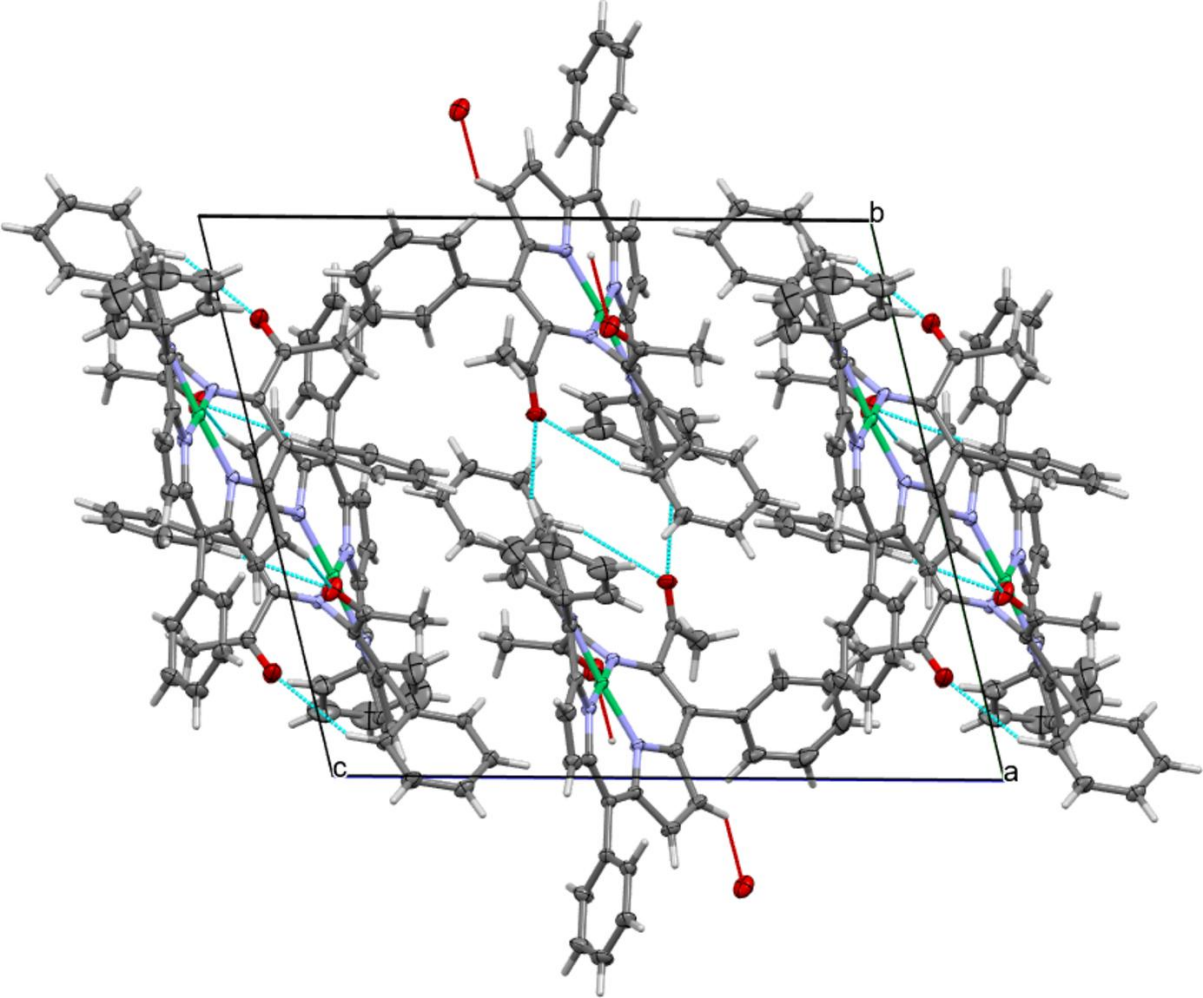
Packing view showing intra- and inter­molecular C—H⋯O hydrogen bonds in **1Ni**. Mol­ecules **1Ni-1** are to the left and right at (*x*, 0, *z*) and (*x*, 1, *z*), mol­ecules **1Ni-2** at the center at (*x*, ½, *z*). Note that all C—H⋯O hydrogen bonds are formed between mol­ecules 1, and between mol­ecules 2, thus forming C—H⋯O bonded layers, while all inter­actions between mol­ecules 1 and 2 are purely van der Waals in nature.

**Figure 6:**
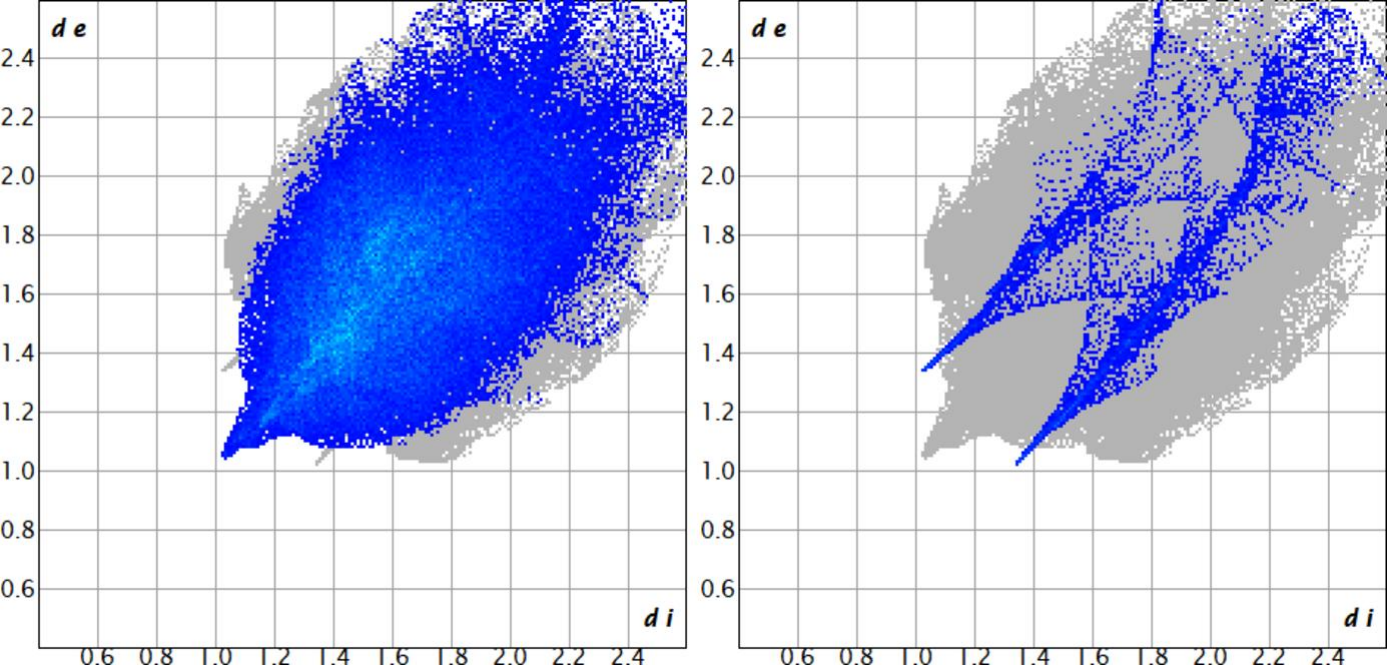
Selected fingerprint plots highlighting the dominance of inter­actions involving H atoms in the structure of **1Ni**. Shown are *d*_e_ *versus d*_i_ plots (*Crystal Explorer*; Spackman *et al.*, 2021 ▸) of H⋯H inter­actions (left) and O⋯H/H⋯O inter­actions (right) for mol­ecule **1Ni-1** (plots for mol­ecule **1Ni-2** are equivalent).

In addition to the intra­molecular C—H⋯O bonds within each mol­ecule, there are also a small number of weak C—H⋯O hydrogen bond-like inter­actions present that connect mol­ecules of **1Ni** with each other (Fig. 5 ▸; see Table 1 ▸ for numerical values and symmetry operators). Some weak C—H⋯π inter­actions are also present (not shown), but these are too weak to be classified as directional and they are unlikely to have any strong structure-determining effects. Instead, inter­molecular inter­actions in the structure of **1Ni** are dominated by non-directional dispersion inter­actions (van der Waals inter­actions). This is qu­anti­tatively confirmed by analysis of the Hirshfeld surfaces of the two mol­ecules (Spackman & Byrom, 1997 ▸; *Crystal Explorer*, Spackman *et al.*, 2021 ▸), which are dominated by C⋯H and H⋯H and reciprocal contacts, making up 23.5 and 59.2% of the Hirshfeld surface of **1Ni-1**, and 23.2 and 58.7% for **1Ni-2**. The shortest contacts are some of the H⋯H contacts, and the few directional C–H⋯O inter­actions (for visualization, see Fig. 6 ▸ showing the *d*_e_ *versus d*_i_ fingerprint plots for H⋯H and H⋯O contacts for **1Ni-1**). N⋯H and Ni⋯H contacts are also observed (5.0 and 2.0% for **1Ni-1**, 4.9% and 2.0% for **1Ni-2**), but distances are rather long and the inter­actions are non-directional. The deformation modes of the mol­ecules also prevent any effective π-stacking inter­actions to be established in the structure, which is confirmed by the low number for C⋯C contacts on the Hirshfeld surface (3.6 and 3.0% for the two mol­ecules).

## 4. Database survey

The structures most closely related to title compound **1Ni** are its aldehyde analogue **5Ni** [CSD (Groom *et al.*, 2016 ▸) code GUBWAB; Brückner *et al.*, 1999 ▸], as well as two related bis-ketone derivatives in which the C atoms of the cleaved pyrrole have been annelated to the adjacent phenyl rings to form an indaphyrin (*meso*-di­phenyl­indaporphyrinato)platinum(II) (CSD entry SUNXAB; Lau *et al.*, 2009 ▸) and its bis­hydroxy­lated indachlorin counterpart (CSD entry OJEHAO; Samankumara *et al.*, 2015 ▸).

## 5. Synthesis and crystallization

The title compound **1Ni** was prepared by classic lead(IV)-induced diol cleavage of *trans-*di­hydroxy­chlorin **2Ni**. This diol was made in two steps from *cis*-diol **3Ni**: oxidation to dione **4Ni** (Daniell *et al.*, 2003 ▸), followed by double methyl-Grignard addition (Banerjee *et al.*, 2012 ▸). Crystals of **1Ni** were grown by slow evaporation of a solution of **1Ni** in CHCl_3_/hexane to dryness. The spectroscopic data of **1Ni** have been reported previously (Banerjee *et al.*, 2012 ▸).

## 6. Refinement

Crystal data, data collection and structure refinement details are summarized in Table 2 ▸. Two crystallographically independent mol­ecules are present in the asymmetric unit of the structure. A common atom-naming scheme combined with residue numbers 1 and 2 were used for the two mol­ecules, which are related by pseudo-A lattice centering. Exact translational symmetry is broken by different torsion angles of one of the acetyl groups (O1, C1, C2), of the adjacent phenyl group C23 to C28), and of the phenyl group trans across the mol­ecule (C35 to C40).

C—H bond distances were constrained to 0.95 Å for aromatic moieties, and to 0.98 Å for CH_3_ moieties. Methyl CH_3_ groups were allowed to rotate but not to tip to best fit the experimental electron density. *U*_iso_(H) values were set to a multiple of *U*_eq_(C) with 1.5 for CH_3_ and 1.2 for C—H units, respectively.

## Supplementary Material

Crystal structure: contains datablock(s) I. DOI: 10.1107/S2056989024004717/yy2012sup1.cif

Structure factors: contains datablock(s) I. DOI: 10.1107/S2056989024004717/yy2012Isup2.hkl

CCDC reference: 2356903

Additional supporting information:  crystallographic information; 3D view; checkCIF report

## Figures and Tables

**Table 1:** Hydrogen-bond geometry (Å, °)

*D*—H⋯*A*	*D*—H	H⋯*A*	*D*⋯*A*	*D*—H⋯*A*
C28_1—H28_1⋯O2_1	0.95	2.60	3.4517 (17)	149
C42_1—H42_1⋯O1_1	0.95	2.55	3.3855 (17)	147
C42_2—H42_2⋯O1_2	0.95	2.66	3.5120 (17)	149
C6_1—H6_1⋯O2_1^i^	0.95	2.49	3.3714 (16)	155
C6_2—H6_2⋯O2_2^ii^	0.95	2.57	3.3569 (17)	140
C17_2—H17_2⋯O1_2^iii^	0.95	2.40	3.2977 (16)	158

Symmetry codes: (i) 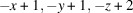 ; (ii) 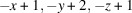 ; (iii) 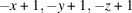 .

**Table 2:** Experimental details

Crystal data	
Chemical formula	[Ni(C_46_H_32_N_4_O_2_)]
*M*_r_	731.46
Crystal system, space group	Triclinic, *P* 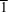
Temperature (K)	150
*a*, *b*, *c* (Å)	14.1382 (5), 15.3202 (5), 18.3835 (7)
α, β, γ (°)	72.0900 (18), 69.6031 (19), 74.6737 (18)
*V* (Å^3^)	3495.8 (2)
*Z*	4
Radiation type	Mo *K*α
μ (mm^−1^)	0.60
Crystal size (mm)	0.24 × 0.16 × 0.14

Data collection	
Diffractometer	Bruker AXS D8 Quest
Absorption correction	Multi-scan (*SADABS*; Krause *et al.*, 2015 ▸)
*T*_min_, *T*_max_	0.701, 0.747
No. of measured, independent and observed [*I* > 2σ(*I*)] reflections	226909, 26754, 19735
*R*_int_	0.056
(sin θ/λ)_max_ (Å^−1^)	0.770

Refinement	
*R*[*F* ^2^ > 2σ(*F* ^2^)], *wR*(*F* ^2^), *S*	0.038, 0.103, 1.01
No. of reflections	26754
No. of parameters	959
H-atom treatment	H-atom parameters constrained
Δρ_max_, Δρ_min_ (e Å^−3^)	1.07, −0.42

Computer programs: *APEX4* (Bruker, 2022 ▸), *SAINT* (Bruker, 2020 ▸), *SHELXT* (Sheldrick, 2015*a* ▸), *SHELXL2019/2* (Sheldrick, 2015*b* ▸), *ShelXle* (Hübschle *et al.*, 2011 ▸), *Mercury* (Macrae *et al.*, 2020 ▸) and *publCIF* (Westrip, 2010 ▸).

## supplementary crystallographic information

### Crystal data

**Table:**

[Ni(C_46_H_32_N_4_O_2_)]	*Z* = 4
*M**_r_* = 731.46	*F*(000) = 1520
Triclinic, *P*1	*D*_x_ = 1.390 Mg m^−^^3^
*a* = 14.1382 (5) Å	Mo *K*α radiation, λ = 0.71073 Å
*b* = 15.3202 (5) Å	Cell parameters from 9410 reflections
*c* = 18.3835 (7) Å	θ = 2.2–33.1°
α = 72.0900 (18)°	µ = 0.60 mm^−^^1^
β = 69.6031 (19)°	*T* = 150 K
γ = 74.6737 (18)°	Block, black
*V* = 3495.8 (2) Å^3^	0.24 × 0.16 × 0.14 mm

### Data collection

**Table:**

Bruker AXS D8 Quest diffractometer	26754 independent reflections
Radiation source: fine focus sealed tube X-ray source	19735 reflections with *I* > 2σ(*I*)
Triumph curved graphite crystal monochromator	*R*_int_ = 0.056
Detector resolution: 7.4074 pixels mm^-1^	θ_max_ = 33.2°, θ_min_ = 1.9°
ω and phi scans	*h* = −21→21
Absorption correction: multi-scan (SADABS; Krause *et al.*, 2015)	*k* = −23→23
*T*_min_ = 0.701, *T*_max_ = 0.747	*l* = −28→28
226909 measured reflections	

### Refinement

**Table:**

Refinement on *F*^2^	Primary atom site location: dual
Least-squares matrix: full	Secondary atom site location: difference Fourier map
*R*[*F*^2^ > 2σ(*F*^2^)] = 0.038	Hydrogen site location: inferred from neighbouring sites
*wR*(*F*^2^) = 0.103	H-atom parameters constrained
*S* = 1.01	*w* = 1/[σ^2^(*F*_o_^2^) + (0.0457*P*)^2^ + 1.6735*P*] where *P* = (*F*_o_^2^ + 2*F*_c_^2^)/3
26754 reflections	(Δ/σ)_max_ = 0.002
959 parameters	Δρ_max_ = 1.07 e Å^−^^3^
0 restraints	Δρ_min_ = −0.42 e Å^−^^3^

### Special details

**Table:**

Geometry. All esds (except the esd in the dihedral angle between two l.s. planes) are estimated using the full covariance matrix. The cell esds are taken into account individually in the estimation of esds in distances, angles and torsion angles; correlations between esds in cell parameters are only used when they are defined by crystal symmetry. An approximate (isotropic) treatment of cell esds is used for estimating esds involving l.s. planes.

### Fractional atomic coordinates and isotropic or equivalent isotropic displacement parameters (Å^2^)

**Table:**

	*x*	*y*	*z*	*U*_iso_*/*U*_eq_	
Ni1_1	0.25969 (2)	0.35659 (2)	0.92873 (2)	0.01429 (4)	
O1_1	0.50774 (9)	0.18303 (8)	1.05354 (7)	0.0319 (2)	
O2_1	0.58223 (8)	0.33407 (9)	0.93406 (7)	0.0312 (2)	
N1_1	0.38355 (8)	0.31234 (7)	0.95721 (6)	0.01569 (18)	
N2_1	0.24377 (8)	0.46934 (7)	0.95719 (6)	0.01615 (18)	
N3_1	0.13540 (8)	0.40147 (8)	0.89948 (6)	0.01684 (19)	
N4_1	0.27362 (8)	0.24007 (7)	0.90697 (6)	0.01638 (18)	
C1_1	0.38050 (13)	0.22630 (10)	1.16637 (9)	0.0284 (3)	
H1A_1	0.309459	0.258994	1.173210	0.043*	
H1B_1	0.413305	0.249917	1.193665	0.043*	
H1C_1	0.381312	0.159369	1.189290	0.043*	
C2_1	0.43835 (11)	0.24268 (9)	1.07820 (8)	0.0209 (2)	
C3_1	0.39427 (9)	0.32810 (9)	1.02306 (7)	0.0165 (2)	
C4_1	0.35152 (9)	0.41150 (9)	1.04696 (7)	0.0165 (2)	
C5_1	0.29060 (9)	0.48299 (9)	1.00558 (7)	0.0164 (2)	
C6_1	0.26648 (10)	0.57957 (9)	1.00854 (8)	0.0196 (2)	
H6_1	0.289412	0.606624	1.038033	0.023*	
C7_1	0.20478 (10)	0.62434 (9)	0.96112 (8)	0.0201 (2)	
H7_1	0.177902	0.689164	0.949856	0.024*	
C8_1	0.18749 (9)	0.55514 (9)	0.93090 (7)	0.0174 (2)	
C9_1	0.11406 (9)	0.56829 (9)	0.89235 (7)	0.0173 (2)	
C10_1	0.08651 (9)	0.49248 (9)	0.88161 (7)	0.0177 (2)	
C11_1	−0.00208 (10)	0.49644 (10)	0.85849 (8)	0.0223 (2)	
H11_1	−0.046378	0.551116	0.840264	0.027*	
C12_1	−0.01033 (10)	0.40691 (10)	0.86772 (8)	0.0228 (2)	
H12_1	−0.063470	0.387129	0.859720	0.027*	
C13_1	0.07627 (10)	0.34801 (9)	0.89178 (8)	0.0189 (2)	
C14_1	0.09973 (10)	0.25128 (10)	0.90294 (8)	0.0202 (2)	
C15_1	0.19504 (10)	0.20055 (9)	0.90892 (8)	0.0192 (2)	
C16_1	0.23266 (11)	0.10522 (9)	0.90465 (9)	0.0233 (3)	
H16_1	0.193543	0.061951	0.907329	0.028*	
C17_1	0.33495 (11)	0.08832 (9)	0.89605 (8)	0.0221 (2)	
H17_1	0.381182	0.031590	0.890142	0.027*	
C18_1	0.35961 (10)	0.17279 (9)	0.89760 (7)	0.0172 (2)	
C19_1	0.45760 (9)	0.18739 (9)	0.88887 (7)	0.0166 (2)	
C20_1	0.46814 (9)	0.25855 (8)	0.91549 (7)	0.0160 (2)	
C21_1	0.56934 (10)	0.29057 (9)	0.89428 (8)	0.0196 (2)	
C22_1	0.65030 (11)	0.27887 (11)	0.81656 (9)	0.0269 (3)	
H22A_1	0.617494	0.276390	0.778420	0.040*	
H22B_1	0.698974	0.220902	0.826505	0.040*	
H22C_1	0.686832	0.331738	0.794441	0.040*	
C23_1	0.38006 (10)	0.42935 (9)	1.11065 (7)	0.0178 (2)	
C24_1	0.30557 (11)	0.45948 (10)	1.17573 (8)	0.0227 (2)	
H24_1	0.235219	0.473075	1.177851	0.027*	
C25_1	0.33437 (13)	0.46950 (11)	1.23718 (9)	0.0294 (3)	
H25_1	0.283517	0.488885	1.281742	0.035*	
C26_1	0.43730 (14)	0.45131 (11)	1.23382 (9)	0.0312 (3)	
H26_1	0.456645	0.457235	1.276426	0.037*	
C27_1	0.51154 (13)	0.42456 (11)	1.16830 (9)	0.0289 (3)	
H27_1	0.582016	0.414031	1.165124	0.035*	
C28_1	0.48303 (11)	0.41309 (10)	1.10711 (8)	0.0223 (2)	
H28_1	0.534221	0.394005	1.062552	0.027*	
C29_1	0.05941 (10)	0.66507 (9)	0.86838 (7)	0.0187 (2)	
C30_1	0.11538 (11)	0.73360 (10)	0.81570 (8)	0.0232 (3)	
H30_1	0.187249	0.717130	0.793280	0.028*	
C31_1	0.06760 (12)	0.82552 (10)	0.79557 (9)	0.0292 (3)	
H31_1	0.106785	0.871457	0.759764	0.035*	
C32_1	−0.03757 (13)	0.85035 (11)	0.82780 (10)	0.0312 (3)	
H32_1	−0.070447	0.913249	0.814379	0.037*	
C33_1	−0.09416 (12)	0.78273 (11)	0.87963 (9)	0.0290 (3)	
H33_1	−0.166139	0.799422	0.901361	0.035*	
C34_1	−0.04645 (10)	0.69064 (10)	0.90010 (8)	0.0233 (3)	
H34_1	−0.085962	0.644895	0.935791	0.028*	
C35_1	0.01980 (11)	0.20077 (10)	0.90774 (9)	0.0251 (3)	
C36_1	−0.04101 (13)	0.16268 (12)	0.98291 (11)	0.0350 (3)	
H36_1	−0.029458	0.167244	1.029413	0.042*	
C37_1	−0.11882 (16)	0.11784 (15)	0.99029 (15)	0.0520 (5)	
H37_1	−0.161147	0.093025	1.041775	0.062*	
C38_1	−0.13451 (18)	0.10941 (17)	0.92327 (18)	0.0633 (7)	
H38_1	−0.186691	0.077627	0.928461	0.076*	
C39_1	−0.0749 (2)	0.1468 (2)	0.84893 (18)	0.0702 (8)	
H39_1	−0.085961	0.140904	0.802706	0.084*	
C40_1	0.00205 (17)	0.19370 (17)	0.84066 (13)	0.0514 (5)	
H40_1	0.042138	0.220664	0.788935	0.062*	
C41_1	0.54657 (10)	0.11626 (9)	0.85978 (7)	0.0174 (2)	
C42_1	0.60649 (11)	0.06204 (9)	0.90959 (8)	0.0223 (2)	
H42_1	0.592115	0.072094	0.961150	0.027*	
C43_1	0.68732 (11)	−0.00675 (10)	0.88368 (9)	0.0263 (3)	
H43_1	0.727588	−0.043938	0.917916	0.032*	
C44_1	0.70950 (11)	−0.02142 (10)	0.80829 (9)	0.0243 (3)	
H44_1	0.765823	−0.067452	0.790439	0.029*	
C45_1	0.64910 (10)	0.03141 (10)	0.75884 (8)	0.0223 (2)	
H45_1	0.663462	0.020808	0.707480	0.027*	
C46_1	0.56776 (10)	0.09969 (9)	0.78457 (8)	0.0200 (2)	
H46_1	0.526294	0.135331	0.750831	0.024*	
Ni1_2	0.28975 (2)	0.83249 (2)	0.43364 (2)	0.01364 (4)	
O1_2	0.50805 (9)	0.64893 (7)	0.56785 (6)	0.0283 (2)	
O2_2	0.62162 (8)	0.80644 (8)	0.42677 (7)	0.0302 (2)	
N1_2	0.41721 (8)	0.78939 (7)	0.45871 (6)	0.01517 (18)	
N2_2	0.27290 (8)	0.94258 (7)	0.46574 (6)	0.01558 (18)	
N3_2	0.16423 (8)	0.87745 (7)	0.40596 (6)	0.01547 (18)	
N4_2	0.30230 (8)	0.71786 (7)	0.40912 (6)	0.01522 (18)	
C1_2	0.58250 (12)	0.75395 (11)	0.59279 (10)	0.0290 (3)	
H1A_2	0.558184	0.739419	0.651304	0.044*	
H1B_2	0.585476	0.820551	0.572148	0.044*	
H1C_2	0.651045	0.717531	0.575406	0.044*	
C2_2	0.50988 (10)	0.72984 (9)	0.56124 (8)	0.0198 (2)	
C3_2	0.43504 (9)	0.80502 (8)	0.52168 (7)	0.0161 (2)	
C4_2	0.38177 (9)	0.88045 (8)	0.55537 (7)	0.0162 (2)	
C5_2	0.31356 (9)	0.95134 (8)	0.52000 (7)	0.0169 (2)	
C6_2	0.27751 (10)	1.04387 (9)	0.53410 (8)	0.0215 (2)	
H6_2	0.292141	1.066722	0.570835	0.026*	
C7_2	0.21887 (10)	1.09181 (9)	0.48497 (8)	0.0216 (2)	
H7_2	0.187965	1.155733	0.478491	0.026*	
C8_2	0.21207 (9)	1.02782 (8)	0.44431 (7)	0.0169 (2)	
C9_2	0.14189 (9)	1.04319 (8)	0.40260 (7)	0.0168 (2)	
C10_2	0.11645 (9)	0.96877 (8)	0.38785 (7)	0.0165 (2)	
C11_2	0.02957 (10)	0.97407 (9)	0.36228 (8)	0.0215 (2)	
H11_2	−0.013479	1.029283	0.343245	0.026*	
C12_2	0.02063 (10)	0.88518 (10)	0.37046 (9)	0.0230 (3)	
H12_2	−0.031828	0.866311	0.360766	0.028*	
C13_2	0.10522 (9)	0.82533 (9)	0.39647 (8)	0.0180 (2)	
C14_2	0.12783 (10)	0.72855 (9)	0.40686 (7)	0.0179 (2)	
C15_2	0.22293 (10)	0.67784 (8)	0.41273 (7)	0.0168 (2)	
C16_2	0.26007 (10)	0.58159 (9)	0.41017 (8)	0.0188 (2)	
H16_2	0.220438	0.537605	0.415128	0.023*	
C17_2	0.36288 (10)	0.56561 (8)	0.39928 (7)	0.0181 (2)	
H17_2	0.409213	0.508973	0.393253	0.022*	
C18_2	0.38782 (9)	0.65083 (8)	0.39867 (7)	0.0157 (2)	
C19_2	0.48674 (9)	0.66652 (8)	0.38682 (7)	0.0162 (2)	
C20_2	0.49964 (9)	0.73750 (8)	0.41204 (7)	0.0158 (2)	
C21_2	0.60192 (10)	0.76690 (9)	0.38690 (8)	0.0190 (2)	
C22_2	0.67802 (11)	0.75820 (11)	0.30658 (9)	0.0270 (3)	
H22A_2	0.734347	0.706390	0.314416	0.040*	
H22B_2	0.705335	0.816194	0.279880	0.040*	
H22C_2	0.643640	0.746389	0.273455	0.040*	
C23_2	0.39074 (10)	0.88720 (9)	0.63182 (8)	0.0201 (2)	
C24_2	0.43383 (13)	0.95701 (11)	0.63622 (10)	0.0304 (3)	
H24_2	0.461139	1.001193	0.589070	0.037*	
C25_2	0.43662 (16)	0.96161 (13)	0.70989 (13)	0.0447 (5)	
H25_2	0.465790	1.009207	0.712757	0.054*	
C26_2	0.39721 (17)	0.89733 (14)	0.77919 (12)	0.0465 (5)	
H26_2	0.398856	0.901333	0.829278	0.056*	
C27_2	0.35584 (16)	0.82798 (14)	0.77513 (10)	0.0419 (4)	
H27_2	0.329646	0.783414	0.822378	0.050*	
C28_2	0.35222 (12)	0.82288 (12)	0.70191 (9)	0.0289 (3)	
H28_2	0.323090	0.774892	0.699672	0.035*	
C29_2	0.08651 (10)	1.14013 (9)	0.38022 (7)	0.0179 (2)	
C30_2	0.14249 (11)	1.21040 (10)	0.33178 (9)	0.0248 (3)	
H30_2	0.215163	1.195838	0.313527	0.030*	
C31_2	0.09302 (13)	1.30144 (10)	0.30996 (10)	0.0315 (3)	
H31_2	0.131933	1.348605	0.276725	0.038*	
C32_2	−0.01331 (13)	1.32372 (10)	0.33665 (10)	0.0311 (3)	
H32_2	−0.047186	1.385792	0.321171	0.037*	
C33_2	−0.06940 (11)	1.25501 (10)	0.38582 (9)	0.0259 (3)	
H33_2	−0.141950	1.270177	0.404883	0.031*	
C34_2	−0.02000 (10)	1.16353 (9)	0.40760 (8)	0.0205 (2)	
H34_2	−0.059200	1.116750	0.441346	0.025*	
C35_2	0.04848 (10)	0.67982 (9)	0.40823 (9)	0.0227 (2)	
C36_2	−0.03873 (12)	0.67244 (12)	0.47409 (11)	0.0331 (3)	
H36_2	−0.047012	0.698605	0.517019	0.040*	
C37_2	−0.11354 (14)	0.62693 (14)	0.47709 (14)	0.0470 (5)	
H37_2	−0.172623	0.621842	0.522162	0.056*	
C38_2	−0.10216 (17)	0.58903 (15)	0.41462 (15)	0.0515 (5)	
H38_2	−0.153674	0.558473	0.416581	0.062*	
C39_2	−0.01596 (19)	0.59565 (16)	0.34947 (14)	0.0533 (6)	
H39_2	−0.008131	0.569213	0.306810	0.064*	
C40_2	0.05973 (15)	0.64087 (14)	0.34582 (11)	0.0388 (4)	
H40_2	0.118940	0.645131	0.300818	0.047*	
C41_2	0.57430 (9)	0.59449 (8)	0.35779 (7)	0.0165 (2)	
C42_2	0.63499 (10)	0.54212 (9)	0.40731 (8)	0.0210 (2)	
H42_2	0.623945	0.556485	0.456686	0.025*	
C43_2	0.71183 (11)	0.46877 (10)	0.38480 (9)	0.0248 (3)	
H43_2	0.752475	0.432818	0.419087	0.030*	
C44_2	0.72899 (11)	0.44820 (10)	0.31237 (9)	0.0243 (3)	
H44_2	0.781599	0.398362	0.296955	0.029*	
C45_2	0.66938 (10)	0.50038 (10)	0.26241 (8)	0.0220 (2)	
H45_2	0.681787	0.486610	0.212556	0.026*	
C46_2	0.59137 (10)	0.57292 (9)	0.28510 (8)	0.0201 (2)	
H46_2	0.549798	0.607702	0.251235	0.024*	

### Atomic displacement parameters (Å^2^)

**Table:**

	*U*^11^	*U*^22^	*U*^33^	*U*^12^	*U*^13^	*U*^23^
Ni1_1	0.01363 (7)	0.01572 (7)	0.01559 (7)	−0.00219 (5)	−0.00567 (5)	−0.00531 (5)
O1_1	0.0388 (6)	0.0289 (5)	0.0262 (5)	0.0111 (5)	−0.0166 (5)	−0.0106 (4)
O2_1	0.0250 (5)	0.0426 (6)	0.0359 (6)	−0.0121 (5)	−0.0064 (4)	−0.0206 (5)
N1_1	0.0157 (4)	0.0169 (5)	0.0168 (4)	−0.0017 (4)	−0.0061 (4)	−0.0067 (4)
N2_1	0.0156 (4)	0.0177 (5)	0.0165 (4)	−0.0019 (4)	−0.0058 (4)	−0.0055 (4)
N3_1	0.0154 (4)	0.0189 (5)	0.0178 (5)	−0.0026 (4)	−0.0064 (4)	−0.0051 (4)
N4_1	0.0165 (4)	0.0180 (5)	0.0172 (4)	−0.0045 (4)	−0.0065 (4)	−0.0047 (4)
C1_1	0.0365 (8)	0.0232 (6)	0.0215 (6)	−0.0060 (6)	−0.0057 (6)	−0.0022 (5)
C2_1	0.0246 (6)	0.0209 (6)	0.0211 (6)	−0.0032 (5)	−0.0109 (5)	−0.0062 (5)
C3_1	0.0165 (5)	0.0189 (5)	0.0170 (5)	−0.0025 (4)	−0.0070 (4)	−0.0063 (4)
C4_1	0.0161 (5)	0.0187 (5)	0.0170 (5)	−0.0030 (4)	−0.0056 (4)	−0.0066 (4)
C5_1	0.0152 (5)	0.0182 (5)	0.0174 (5)	−0.0028 (4)	−0.0047 (4)	−0.0069 (4)
C6_1	0.0194 (6)	0.0192 (5)	0.0230 (6)	−0.0040 (4)	−0.0057 (5)	−0.0089 (5)
C7_1	0.0201 (6)	0.0175 (5)	0.0233 (6)	−0.0017 (4)	−0.0064 (5)	−0.0067 (5)
C8_1	0.0166 (5)	0.0178 (5)	0.0180 (5)	−0.0016 (4)	−0.0052 (4)	−0.0055 (4)
C9_1	0.0152 (5)	0.0193 (5)	0.0161 (5)	−0.0011 (4)	−0.0043 (4)	−0.0043 (4)
C10_1	0.0151 (5)	0.0214 (6)	0.0170 (5)	−0.0019 (4)	−0.0058 (4)	−0.0049 (4)
C11_1	0.0174 (5)	0.0270 (6)	0.0244 (6)	−0.0007 (5)	−0.0106 (5)	−0.0065 (5)
C12_1	0.0174 (6)	0.0284 (7)	0.0263 (6)	−0.0039 (5)	−0.0094 (5)	−0.0083 (5)
C13_1	0.0161 (5)	0.0234 (6)	0.0191 (5)	−0.0055 (4)	−0.0058 (4)	−0.0054 (5)
C14_1	0.0194 (6)	0.0238 (6)	0.0209 (6)	−0.0084 (5)	−0.0065 (5)	−0.0054 (5)
C15_1	0.0198 (6)	0.0205 (6)	0.0198 (5)	−0.0058 (5)	−0.0072 (4)	−0.0047 (4)
C16_1	0.0274 (7)	0.0187 (6)	0.0283 (6)	−0.0081 (5)	−0.0111 (5)	−0.0051 (5)
C17_1	0.0255 (6)	0.0175 (6)	0.0268 (6)	−0.0040 (5)	−0.0100 (5)	−0.0071 (5)
C18_1	0.0194 (5)	0.0166 (5)	0.0177 (5)	−0.0025 (4)	−0.0076 (4)	−0.0051 (4)
C19_1	0.0178 (5)	0.0170 (5)	0.0166 (5)	−0.0009 (4)	−0.0070 (4)	−0.0058 (4)
C20_1	0.0158 (5)	0.0176 (5)	0.0161 (5)	−0.0010 (4)	−0.0061 (4)	−0.0059 (4)
C21_1	0.0166 (5)	0.0194 (6)	0.0241 (6)	−0.0018 (4)	−0.0075 (5)	−0.0064 (5)
C22_1	0.0227 (6)	0.0265 (7)	0.0291 (7)	−0.0075 (5)	0.0000 (5)	−0.0096 (5)
C23_1	0.0223 (6)	0.0178 (5)	0.0167 (5)	−0.0037 (4)	−0.0074 (4)	−0.0066 (4)
C24_1	0.0266 (6)	0.0232 (6)	0.0198 (6)	−0.0042 (5)	−0.0051 (5)	−0.0089 (5)
C25_1	0.0403 (8)	0.0306 (7)	0.0204 (6)	−0.0066 (6)	−0.0069 (6)	−0.0124 (5)
C26_1	0.0472 (9)	0.0314 (7)	0.0254 (7)	−0.0092 (7)	−0.0189 (6)	−0.0098 (6)
C27_1	0.0329 (7)	0.0338 (8)	0.0298 (7)	−0.0079 (6)	−0.0166 (6)	−0.0108 (6)
C28_1	0.0230 (6)	0.0264 (6)	0.0222 (6)	−0.0061 (5)	−0.0085 (5)	−0.0088 (5)
C29_1	0.0177 (5)	0.0197 (5)	0.0176 (5)	0.0008 (4)	−0.0065 (4)	−0.0047 (4)
C30_1	0.0211 (6)	0.0218 (6)	0.0237 (6)	−0.0021 (5)	−0.0067 (5)	−0.0024 (5)
C31_1	0.0314 (7)	0.0227 (6)	0.0295 (7)	−0.0041 (6)	−0.0119 (6)	0.0024 (5)
C32_1	0.0325 (8)	0.0223 (7)	0.0348 (8)	0.0043 (6)	−0.0155 (6)	−0.0024 (6)
C33_1	0.0226 (6)	0.0293 (7)	0.0299 (7)	0.0058 (5)	−0.0086 (5)	−0.0074 (6)
C34_1	0.0198 (6)	0.0252 (6)	0.0208 (6)	0.0008 (5)	−0.0057 (5)	−0.0043 (5)
C35_1	0.0214 (6)	0.0253 (6)	0.0341 (7)	−0.0075 (5)	−0.0114 (5)	−0.0078 (5)
C36_1	0.0337 (8)	0.0299 (8)	0.0424 (9)	−0.0146 (6)	−0.0076 (7)	−0.0063 (7)
C37_1	0.0419 (10)	0.0411 (10)	0.0732 (15)	−0.0254 (9)	−0.0101 (10)	−0.0063 (10)
C38_1	0.0483 (12)	0.0566 (13)	0.103 (2)	−0.0304 (11)	−0.0321 (13)	−0.0149 (13)
C39_1	0.0726 (17)	0.092 (2)	0.0810 (18)	−0.0400 (15)	−0.0403 (15)	−0.0267 (15)
C40_1	0.0539 (12)	0.0751 (15)	0.0450 (11)	−0.0336 (11)	−0.0198 (9)	−0.0167 (10)
C41_1	0.0179 (5)	0.0162 (5)	0.0190 (5)	−0.0013 (4)	−0.0066 (4)	−0.0055 (4)
C42_1	0.0258 (6)	0.0217 (6)	0.0228 (6)	0.0020 (5)	−0.0131 (5)	−0.0085 (5)
C43_1	0.0265 (7)	0.0230 (6)	0.0333 (7)	0.0052 (5)	−0.0168 (6)	−0.0108 (5)
C44_1	0.0203 (6)	0.0223 (6)	0.0307 (7)	0.0014 (5)	−0.0073 (5)	−0.0116 (5)
C45_1	0.0220 (6)	0.0227 (6)	0.0210 (6)	−0.0013 (5)	−0.0040 (5)	−0.0084 (5)
C46_1	0.0214 (6)	0.0199 (6)	0.0189 (5)	−0.0004 (5)	−0.0072 (5)	−0.0058 (4)
Ni1_2	0.01412 (7)	0.01168 (7)	0.01588 (7)	0.00009 (5)	−0.00590 (5)	−0.00456 (5)
O1_2	0.0418 (6)	0.0154 (4)	0.0287 (5)	0.0027 (4)	−0.0177 (5)	−0.0046 (4)
O2_2	0.0261 (5)	0.0371 (6)	0.0332 (6)	−0.0126 (4)	−0.0036 (4)	−0.0162 (5)
N1_2	0.0159 (4)	0.0135 (4)	0.0170 (4)	−0.0003 (3)	−0.0063 (4)	−0.0051 (3)
N2_2	0.0166 (4)	0.0133 (4)	0.0173 (4)	0.0000 (3)	−0.0062 (4)	−0.0050 (4)
N3_2	0.0151 (4)	0.0143 (4)	0.0173 (4)	−0.0007 (3)	−0.0056 (4)	−0.0045 (4)
N4_2	0.0157 (4)	0.0131 (4)	0.0171 (4)	−0.0002 (3)	−0.0057 (4)	−0.0049 (3)
C1_2	0.0254 (7)	0.0317 (7)	0.0349 (8)	0.0023 (6)	−0.0180 (6)	−0.0104 (6)
C2_2	0.0214 (6)	0.0185 (5)	0.0187 (5)	0.0013 (4)	−0.0083 (5)	−0.0046 (4)
C3_2	0.0176 (5)	0.0138 (5)	0.0174 (5)	−0.0017 (4)	−0.0063 (4)	−0.0038 (4)
C4_2	0.0180 (5)	0.0151 (5)	0.0177 (5)	−0.0024 (4)	−0.0072 (4)	−0.0052 (4)
C5_2	0.0176 (5)	0.0148 (5)	0.0197 (5)	−0.0009 (4)	−0.0069 (4)	−0.0061 (4)
C6_2	0.0228 (6)	0.0177 (6)	0.0290 (6)	0.0015 (5)	−0.0112 (5)	−0.0123 (5)
C7_2	0.0223 (6)	0.0152 (5)	0.0291 (6)	0.0011 (4)	−0.0100 (5)	−0.0089 (5)
C8_2	0.0164 (5)	0.0134 (5)	0.0194 (5)	0.0006 (4)	−0.0053 (4)	−0.0046 (4)
C9_2	0.0164 (5)	0.0149 (5)	0.0163 (5)	0.0002 (4)	−0.0041 (4)	−0.0032 (4)
C10_2	0.0160 (5)	0.0157 (5)	0.0164 (5)	0.0000 (4)	−0.0055 (4)	−0.0033 (4)
C11_2	0.0198 (6)	0.0208 (6)	0.0248 (6)	0.0022 (5)	−0.0116 (5)	−0.0060 (5)
C12_2	0.0191 (6)	0.0245 (6)	0.0290 (7)	0.0000 (5)	−0.0124 (5)	−0.0085 (5)
C13_2	0.0166 (5)	0.0185 (5)	0.0200 (5)	−0.0020 (4)	−0.0066 (4)	−0.0059 (4)
C14_2	0.0175 (5)	0.0185 (5)	0.0191 (5)	−0.0030 (4)	−0.0060 (4)	−0.0057 (4)
C15_2	0.0190 (5)	0.0154 (5)	0.0171 (5)	−0.0037 (4)	−0.0054 (4)	−0.0048 (4)
C16_2	0.0233 (6)	0.0151 (5)	0.0193 (5)	−0.0040 (4)	−0.0068 (5)	−0.0046 (4)
C17_2	0.0239 (6)	0.0140 (5)	0.0174 (5)	−0.0015 (4)	−0.0072 (4)	−0.0055 (4)
C18_2	0.0188 (5)	0.0136 (5)	0.0159 (5)	−0.0001 (4)	−0.0067 (4)	−0.0053 (4)
C19_2	0.0175 (5)	0.0153 (5)	0.0154 (5)	0.0013 (4)	−0.0063 (4)	−0.0052 (4)
C20_2	0.0156 (5)	0.0148 (5)	0.0170 (5)	0.0006 (4)	−0.0065 (4)	−0.0046 (4)
C21_2	0.0181 (5)	0.0162 (5)	0.0215 (6)	−0.0010 (4)	−0.0060 (4)	−0.0041 (4)
C22_2	0.0233 (6)	0.0320 (7)	0.0228 (6)	−0.0079 (6)	−0.0012 (5)	−0.0064 (5)
C23_2	0.0214 (6)	0.0198 (6)	0.0231 (6)	0.0018 (4)	−0.0112 (5)	−0.0099 (5)
C24_2	0.0387 (8)	0.0216 (6)	0.0416 (8)	−0.0015 (6)	−0.0247 (7)	−0.0105 (6)
C25_2	0.0596 (12)	0.0327 (9)	0.0645 (12)	0.0075 (8)	−0.0443 (11)	−0.0262 (9)
C26_2	0.0631 (13)	0.0490 (11)	0.0394 (9)	0.0152 (9)	−0.0348 (9)	−0.0254 (8)
C27_2	0.0516 (11)	0.0508 (11)	0.0225 (7)	0.0007 (9)	−0.0145 (7)	−0.0114 (7)
C28_2	0.0321 (7)	0.0357 (8)	0.0205 (6)	−0.0070 (6)	−0.0076 (5)	−0.0078 (6)
C29_2	0.0191 (5)	0.0152 (5)	0.0162 (5)	0.0012 (4)	−0.0051 (4)	−0.0030 (4)
C30_2	0.0208 (6)	0.0200 (6)	0.0263 (6)	−0.0017 (5)	−0.0039 (5)	−0.0003 (5)
C31_2	0.0318 (8)	0.0179 (6)	0.0344 (8)	−0.0028 (5)	−0.0073 (6)	0.0041 (5)
C32_2	0.0331 (8)	0.0181 (6)	0.0351 (8)	0.0052 (5)	−0.0138 (6)	−0.0007 (6)
C33_2	0.0222 (6)	0.0210 (6)	0.0293 (7)	0.0059 (5)	−0.0081 (5)	−0.0061 (5)
C34_2	0.0188 (6)	0.0189 (6)	0.0190 (5)	0.0007 (4)	−0.0042 (4)	−0.0027 (4)
C35_2	0.0208 (6)	0.0199 (6)	0.0310 (7)	−0.0051 (5)	−0.0114 (5)	−0.0053 (5)
C36_2	0.0245 (7)	0.0303 (8)	0.0442 (9)	−0.0082 (6)	−0.0039 (6)	−0.0126 (7)
C37_2	0.0272 (8)	0.0414 (10)	0.0714 (14)	−0.0150 (7)	−0.0060 (8)	−0.0138 (9)
C38_2	0.0454 (11)	0.0474 (11)	0.0769 (15)	−0.0252 (9)	−0.0286 (11)	−0.0092 (10)
C39_2	0.0707 (15)	0.0594 (13)	0.0539 (12)	−0.0339 (12)	−0.0297 (11)	−0.0144 (10)
C40_2	0.0469 (10)	0.0483 (10)	0.0332 (8)	−0.0235 (8)	−0.0128 (7)	−0.0122 (7)
C41_2	0.0169 (5)	0.0164 (5)	0.0165 (5)	0.0011 (4)	−0.0062 (4)	−0.0063 (4)
C42_2	0.0234 (6)	0.0205 (6)	0.0201 (6)	0.0032 (5)	−0.0098 (5)	−0.0084 (5)
C43_2	0.0239 (6)	0.0244 (6)	0.0268 (6)	0.0074 (5)	−0.0136 (5)	−0.0098 (5)
C44_2	0.0204 (6)	0.0242 (6)	0.0289 (7)	0.0049 (5)	−0.0077 (5)	−0.0139 (5)
C45_2	0.0203 (6)	0.0269 (6)	0.0198 (6)	0.0001 (5)	−0.0043 (5)	−0.0122 (5)
C46_2	0.0197 (6)	0.0225 (6)	0.0183 (5)	0.0019 (5)	−0.0069 (4)	−0.0085 (5)

### Geometric parameters (Å, º)

**Table:**

Ni1_1—N2_1	1.8911 (11)	Ni1_2—N2_2	1.8845 (10)
Ni1_1—N4_1	1.8930 (11)	Ni1_2—N4_2	1.8934 (10)
Ni1_1—N1_1	1.8957 (10)	Ni1_2—N3_2	1.9106 (10)
Ni1_1—N3_1	1.9084 (11)	Ni1_2—N1_2	1.9128 (10)
O1_1—C2_1	1.2141 (17)	O1_2—C2_2	1.2141 (16)
O2_1—C21_1	1.2122 (16)	O2_2—C21_2	1.2192 (16)
N1_1—C3_1	1.3687 (15)	N1_2—C3_2	1.3661 (15)
N1_1—C20_1	1.3879 (15)	N1_2—C20_2	1.3997 (15)
N2_1—C5_1	1.3680 (15)	N2_2—C5_2	1.3661 (16)
N2_1—C8_1	1.3853 (16)	N2_2—C8_2	1.3880 (15)
N3_1—C10_1	1.3772 (16)	N3_2—C10_2	1.3766 (15)
N3_1—C13_1	1.3804 (16)	N3_2—C13_2	1.3796 (16)
N4_1—C18_1	1.3678 (16)	N4_2—C18_2	1.3626 (15)
N4_1—C15_1	1.3844 (16)	N4_2—C15_2	1.3879 (16)
C1_1—C2_1	1.514 (2)	C1_2—C2_2	1.508 (2)
C1_1—H1A_1	0.9800	C1_2—H1A_2	0.9800
C1_1—H1B_1	0.9800	C1_2—H1B_2	0.9800
C1_1—H1C_1	0.9800	C1_2—H1C_2	0.9800
C2_1—C3_1	1.5239 (18)	C2_2—C3_2	1.5336 (17)
C3_1—C4_1	1.3938 (17)	C3_2—C4_2	1.3934 (17)
C4_1—C5_1	1.4022 (17)	C4_2—C5_2	1.4075 (17)
C4_1—C23_1	1.4808 (16)	C4_2—C23_2	1.4908 (17)
C5_1—C6_1	1.4417 (17)	C5_2—C6_2	1.4452 (17)
C6_1—C7_1	1.3556 (19)	C6_2—C7_2	1.3544 (19)
C6_1—H6_1	0.9500	C6_2—H6_2	0.9500
C7_1—C8_1	1.4442 (18)	C7_2—C8_2	1.4420 (18)
C7_1—H7_1	0.9500	C7_2—H7_2	0.9500
C8_1—C9_1	1.3912 (17)	C8_2—C9_2	1.3870 (17)
C9_1—C10_1	1.4048 (18)	C9_2—C10_2	1.4063 (17)
C9_1—C29_1	1.4898 (18)	C9_2—C29_2	1.4906 (17)
C10_1—C11_1	1.4381 (17)	C10_2—C11_2	1.4362 (18)
C11_1—C12_1	1.361 (2)	C11_2—C12_2	1.3583 (19)
C11_1—H11_1	0.9500	C11_2—H11_2	0.9500
C12_1—C13_1	1.4342 (19)	C12_2—C13_2	1.4315 (18)
C12_1—H12_1	0.9500	C12_2—H12_2	0.9500
C13_1—C14_1	1.3960 (19)	C13_2—C14_2	1.3981 (18)
C14_1—C15_1	1.3889 (19)	C14_2—C15_2	1.3858 (18)
C14_1—C35_1	1.4948 (18)	C14_2—C35_2	1.4927 (18)
C15_1—C16_1	1.4328 (19)	C15_2—C16_2	1.4383 (17)
C16_1—C17_1	1.361 (2)	C16_2—C17_2	1.3609 (18)
C16_1—H16_1	0.9500	C16_2—H16_2	0.9500
C17_1—C18_1	1.4371 (17)	C17_2—C18_2	1.4355 (17)
C17_1—H17_1	0.9500	C17_2—H17_2	0.9500
C18_1—C19_1	1.4078 (17)	C18_2—C19_2	1.4121 (17)
C19_1—C20_1	1.3839 (17)	C19_2—C20_2	1.3801 (17)
C19_1—C41_1	1.4902 (17)	C19_2—C41_2	1.4881 (16)
C20_1—C21_1	1.5179 (17)	C20_2—C21_2	1.5027 (18)
C21_1—C22_1	1.5142 (19)	C21_2—C22_2	1.5145 (19)
C22_1—H22A_1	0.9800	C22_2—H22A_2	0.9800
C22_1—H22B_1	0.9800	C22_2—H22B_2	0.9800
C22_1—H22C_1	0.9800	C22_2—H22C_2	0.9800
C23_1—C28_1	1.3922 (18)	C23_2—C28_2	1.393 (2)
C23_1—C24_1	1.3992 (18)	C23_2—C24_2	1.3977 (19)
C24_1—C25_1	1.3878 (19)	C24_2—C25_2	1.392 (2)
C24_1—H24_1	0.9500	C24_2—H24_2	0.9500
C25_1—C26_1	1.390 (2)	C25_2—C26_2	1.388 (3)
C25_1—H25_1	0.9500	C25_2—H25_2	0.9500
C26_1—C27_1	1.384 (2)	C26_2—C27_2	1.373 (3)
C26_1—H26_1	0.9500	C26_2—H26_2	0.9500
C27_1—C28_1	1.3906 (19)	C27_2—C28_2	1.391 (2)
C27_1—H27_1	0.9500	C27_2—H27_2	0.9500
C28_1—H28_1	0.9500	C28_2—H28_2	0.9500
C29_1—C30_1	1.3950 (19)	C29_2—C34_2	1.3952 (18)
C29_1—C34_1	1.3985 (18)	C29_2—C30_2	1.3957 (18)
C30_1—C31_1	1.388 (2)	C30_2—C31_2	1.390 (2)
C30_1—H30_1	0.9500	C30_2—H30_2	0.9500
C31_1—C32_1	1.390 (2)	C31_2—C32_2	1.391 (2)
C31_1—H31_1	0.9500	C31_2—H31_2	0.9500
C32_1—C33_1	1.386 (2)	C32_2—C33_2	1.382 (2)
C32_1—H32_1	0.9500	C32_2—H32_2	0.9500
C33_1—C34_1	1.391 (2)	C33_2—C34_2	1.3944 (18)
C33_1—H33_1	0.9500	C33_2—H33_2	0.9500
C34_1—H34_1	0.9500	C34_2—H34_2	0.9500
C35_1—C40_1	1.381 (2)	C35_2—C40_2	1.393 (2)
C35_1—C36_1	1.389 (2)	C35_2—C36_2	1.396 (2)
C36_1—C37_1	1.392 (2)	C36_2—C37_2	1.390 (2)
C36_1—H36_1	0.9500	C36_2—H36_2	0.9500
C37_1—C38_1	1.374 (4)	C37_2—C38_2	1.383 (3)
C37_1—H37_1	0.9500	C37_2—H37_2	0.9500
C38_1—C39_1	1.370 (4)	C38_2—C39_2	1.380 (3)
C38_1—H38_1	0.9500	C38_2—H38_2	0.9500
C39_1—C40_1	1.397 (3)	C39_2—C40_2	1.394 (2)
C39_1—H39_1	0.9500	C39_2—H39_2	0.9500
C40_1—H40_1	0.9500	C40_2—H40_2	0.9500
C41_1—C42_1	1.3953 (18)	C41_2—C42_2	1.3928 (17)
C41_1—C46_1	1.3975 (17)	C41_2—C46_2	1.3993 (17)
C42_1—C43_1	1.3915 (19)	C42_2—C43_2	1.3934 (18)
C42_1—H42_1	0.9500	C42_2—H42_2	0.9500
C43_1—C44_1	1.386 (2)	C43_2—C44_2	1.3869 (19)
C43_1—H43_1	0.9500	C43_2—H43_2	0.9500
C44_1—C45_1	1.391 (2)	C44_2—C45_2	1.387 (2)
C44_1—H44_1	0.9500	C44_2—H44_2	0.9500
C45_1—C46_1	1.3891 (18)	C45_2—C46_2	1.3935 (18)
C45_1—H45_1	0.9500	C45_2—H45_2	0.9500
C46_1—H46_1	0.9500	C46_2—H46_2	0.9500
			
N2_1—Ni1_1—N4_1	176.50 (5)	N2_2—Ni1_2—N4_2	175.90 (5)
N2_1—Ni1_1—N1_1	90.06 (4)	N2_2—Ni1_2—N3_2	90.19 (4)
N4_1—Ni1_1—N1_1	88.86 (4)	N4_2—Ni1_2—N3_2	90.20 (4)
N2_1—Ni1_1—N3_1	89.92 (5)	N2_2—Ni1_2—N1_2	89.59 (4)
N4_1—Ni1_1—N3_1	91.19 (5)	N4_2—Ni1_2—N1_2	90.12 (4)
N1_1—Ni1_1—N3_1	179.68 (5)	N3_2—Ni1_2—N1_2	178.50 (5)
C3_1—N1_1—C20_1	114.99 (10)	C3_2—N1_2—C20_2	115.41 (10)
C3_1—N1_1—Ni1_1	121.95 (8)	C3_2—N1_2—Ni1_2	124.13 (8)
C20_1—N1_1—Ni1_1	123.00 (8)	C20_2—N1_2—Ni1_2	120.46 (8)
C5_1—N2_1—C8_1	106.09 (10)	C5_2—N2_2—C8_2	106.48 (10)
C5_1—N2_1—Ni1_1	126.12 (9)	C5_2—N2_2—Ni1_2	125.72 (8)
C8_1—N2_1—Ni1_1	127.72 (8)	C8_2—N2_2—Ni1_2	127.65 (8)
C10_1—N3_1—C13_1	106.00 (10)	C10_2—N3_2—C13_2	105.79 (10)
C10_1—N3_1—Ni1_1	127.71 (9)	C10_2—N3_2—Ni1_2	127.17 (8)
C13_1—N3_1—Ni1_1	126.29 (9)	C13_2—N3_2—Ni1_2	126.99 (8)
C18_1—N4_1—C15_1	106.00 (10)	C18_2—N4_2—C15_2	105.74 (10)
C18_1—N4_1—Ni1_1	127.08 (8)	C18_2—N4_2—Ni1_2	126.63 (8)
C15_1—N4_1—Ni1_1	126.32 (9)	C15_2—N4_2—Ni1_2	126.67 (8)
C2_1—C1_1—H1A_1	109.5	C2_2—C1_2—H1A_2	109.5
C2_1—C1_1—H1B_1	109.5	C2_2—C1_2—H1B_2	109.5
H1A_1—C1_1—H1B_1	109.5	H1A_2—C1_2—H1B_2	109.5
C2_1—C1_1—H1C_1	109.5	C2_2—C1_2—H1C_2	109.5
H1A_1—C1_1—H1C_1	109.5	H1A_2—C1_2—H1C_2	109.5
H1B_1—C1_1—H1C_1	109.5	H1B_2—C1_2—H1C_2	109.5
O1_1—C2_1—C1_1	119.75 (13)	O1_2—C2_2—C1_2	120.23 (12)
O1_1—C2_1—C3_1	122.85 (12)	O1_2—C2_2—C3_2	117.78 (12)
C1_1—C2_1—C3_1	116.68 (12)	C1_2—C2_2—C3_2	121.98 (12)
N1_1—C3_1—C4_1	122.96 (11)	N1_2—C3_2—C4_2	123.14 (11)
N1_1—C3_1—C2_1	115.49 (11)	N1_2—C3_2—C2_2	117.19 (10)
C4_1—C3_1—C2_1	120.56 (11)	C4_2—C3_2—C2_2	119.38 (11)
C3_1—C4_1—C5_1	120.68 (11)	C3_2—C4_2—C5_2	120.86 (11)
C3_1—C4_1—C23_1	119.78 (11)	C3_2—C4_2—C23_2	121.66 (11)
C5_1—C4_1—C23_1	119.17 (11)	C5_2—C4_2—C23_2	117.42 (11)
N2_1—C5_1—C4_1	123.75 (11)	N2_2—C5_2—C4_2	124.37 (11)
N2_1—C5_1—C6_1	110.40 (11)	N2_2—C5_2—C6_2	109.94 (11)
C4_1—C5_1—C6_1	125.84 (11)	C4_2—C5_2—C6_2	125.68 (11)
C7_1—C6_1—C5_1	106.76 (11)	C7_2—C6_2—C5_2	106.94 (11)
C7_1—C6_1—H6_1	126.6	C7_2—C6_2—H6_2	126.5
C5_1—C6_1—H6_1	126.6	C5_2—C6_2—H6_2	126.5
C6_1—C7_1—C8_1	107.26 (11)	C6_2—C7_2—C8_2	107.30 (11)
C6_1—C7_1—H7_1	126.4	C6_2—C7_2—H7_2	126.4
C8_1—C7_1—H7_1	126.4	C8_2—C7_2—H7_2	126.4
N2_1—C8_1—C9_1	124.27 (11)	C9_2—C8_2—N2_2	124.72 (11)
N2_1—C8_1—C7_1	109.40 (11)	C9_2—C8_2—C7_2	125.10 (11)
C9_1—C8_1—C7_1	125.61 (12)	N2_2—C8_2—C7_2	109.18 (11)
C8_1—C9_1—C10_1	121.36 (12)	C8_2—C9_2—C10_2	121.26 (11)
C8_1—C9_1—C29_1	117.74 (11)	C8_2—C9_2—C29_2	118.14 (11)
C10_1—C9_1—C29_1	120.73 (11)	C10_2—C9_2—C29_2	120.36 (11)
N3_1—C10_1—C9_1	124.35 (11)	N3_2—C10_2—C9_2	124.11 (11)
N3_1—C10_1—C11_1	109.92 (11)	N3_2—C10_2—C11_2	109.93 (11)
C9_1—C10_1—C11_1	125.44 (12)	C9_2—C10_2—C11_2	125.47 (11)
C12_1—C11_1—C10_1	106.90 (12)	C12_2—C11_2—C10_2	107.00 (11)
C12_1—C11_1—H11_1	126.6	C12_2—C11_2—H11_2	126.5
C10_1—C11_1—H11_1	126.6	C10_2—C11_2—H11_2	126.5
C11_1—C12_1—C13_1	107.12 (12)	C11_2—C12_2—C13_2	107.00 (12)
C11_1—C12_1—H12_1	126.4	C11_2—C12_2—H12_2	126.5
C13_1—C12_1—H12_1	126.4	C13_2—C12_2—H12_2	126.5
N3_1—C13_1—C14_1	124.80 (12)	N3_2—C13_2—C14_2	124.81 (11)
N3_1—C13_1—C12_1	109.91 (11)	N3_2—C13_2—C12_2	110.12 (11)
C14_1—C13_1—C12_1	125.25 (12)	C14_2—C13_2—C12_2	125.00 (12)
C15_1—C14_1—C13_1	122.27 (12)	C15_2—C14_2—C13_2	121.64 (11)
C15_1—C14_1—C35_1	118.93 (12)	C15_2—C14_2—C35_2	119.60 (11)
C13_1—C14_1—C35_1	118.80 (12)	C13_2—C14_2—C35_2	118.71 (11)
N4_1—C15_1—C14_1	123.63 (12)	C14_2—C15_2—N4_2	123.45 (11)
N4_1—C15_1—C16_1	109.74 (11)	C14_2—C15_2—C16_2	126.11 (11)
C14_1—C15_1—C16_1	125.89 (12)	N4_2—C15_2—C16_2	109.72 (11)
C17_1—C16_1—C15_1	107.11 (11)	C17_2—C16_2—C15_2	106.83 (11)
C17_1—C16_1—H16_1	126.4	C17_2—C16_2—H16_2	126.6
C15_1—C16_1—H16_1	126.4	C15_2—C16_2—H16_2	126.6
C16_1—C17_1—C18_1	106.80 (12)	C16_2—C17_2—C18_2	106.79 (11)
C16_1—C17_1—H17_1	126.6	C16_2—C17_2—H17_2	126.6
C18_1—C17_1—H17_1	126.6	C18_2—C17_2—H17_2	126.6
N4_1—C18_1—C19_1	123.53 (11)	N4_2—C18_2—C19_2	123.16 (11)
N4_1—C18_1—C17_1	110.26 (11)	N4_2—C18_2—C17_2	110.73 (11)
C19_1—C18_1—C17_1	126.19 (12)	C19_2—C18_2—C17_2	126.08 (11)
C20_1—C19_1—C18_1	120.46 (11)	C20_2—C19_2—C18_2	120.95 (11)
C20_1—C19_1—C41_1	122.21 (11)	C20_2—C19_2—C41_2	122.01 (11)
C18_1—C19_1—C41_1	116.89 (11)	C18_2—C19_2—C41_2	116.37 (11)
C19_1—C20_1—N1_1	121.39 (11)	C19_2—C20_2—N1_2	121.86 (11)
C19_1—C20_1—C21_1	123.37 (11)	C19_2—C20_2—C21_2	121.72 (11)
N1_1—C20_1—C21_1	114.92 (10)	N1_2—C20_2—C21_2	116.33 (10)
O2_1—C21_1—C22_1	119.18 (12)	O2_2—C21_2—C20_2	120.92 (12)
O2_1—C21_1—C20_1	121.03 (12)	O2_2—C21_2—C22_2	118.27 (12)
C22_1—C21_1—C20_1	119.32 (11)	C20_2—C21_2—C22_2	120.43 (11)
C21_1—C22_1—H22A_1	109.5	C21_2—C22_2—H22A_2	109.5
C21_1—C22_1—H22B_1	109.5	C21_2—C22_2—H22B_2	109.5
H22A_1—C22_1—H22B_1	109.5	H22A_2—C22_2—H22B_2	109.5
C21_1—C22_1—H22C_1	109.5	C21_2—C22_2—H22C_2	109.5
H22A_1—C22_1—H22C_1	109.5	H22A_2—C22_2—H22C_2	109.5
H22B_1—C22_1—H22C_1	109.5	H22B_2—C22_2—H22C_2	109.5
C28_1—C23_1—C24_1	119.16 (12)	C28_2—C23_2—C24_2	118.74 (13)
C28_1—C23_1—C4_1	119.38 (11)	C28_2—C23_2—C4_2	118.70 (12)
C24_1—C23_1—C4_1	121.43 (12)	C24_2—C23_2—C4_2	122.54 (13)
C25_1—C24_1—C23_1	120.05 (14)	C25_2—C24_2—C23_2	119.80 (16)
C25_1—C24_1—H24_1	120.0	C25_2—C24_2—H24_2	120.1
C23_1—C24_1—H24_1	120.0	C23_2—C24_2—H24_2	120.1
C24_1—C25_1—C26_1	120.31 (14)	C26_2—C25_2—C24_2	120.66 (17)
C24_1—C25_1—H25_1	119.8	C26_2—C25_2—H25_2	119.7
C26_1—C25_1—H25_1	119.8	C24_2—C25_2—H25_2	119.7
C27_1—C26_1—C25_1	119.88 (13)	C27_2—C26_2—C25_2	119.79 (16)
C27_1—C26_1—H26_1	120.1	C27_2—C26_2—H26_2	120.1
C25_1—C26_1—H26_1	120.1	C25_2—C26_2—H26_2	120.1
C26_1—C27_1—C28_1	120.04 (14)	C26_2—C27_2—C28_2	120.08 (18)
C26_1—C27_1—H27_1	120.0	C26_2—C27_2—H27_2	120.0
C28_1—C27_1—H27_1	120.0	C28_2—C27_2—H27_2	120.0
C27_1—C28_1—C23_1	120.51 (13)	C27_2—C28_2—C23_2	120.91 (16)
C27_1—C28_1—H28_1	119.7	C27_2—C28_2—H28_2	119.5
C23_1—C28_1—H28_1	119.7	C23_2—C28_2—H28_2	119.5
C30_1—C29_1—C34_1	118.59 (12)	C34_2—C29_2—C30_2	118.63 (12)
C30_1—C29_1—C9_1	119.49 (11)	C34_2—C29_2—C9_2	122.00 (11)
C34_1—C29_1—C9_1	121.85 (12)	C30_2—C29_2—C9_2	119.35 (11)
C31_1—C30_1—C29_1	120.97 (13)	C31_2—C30_2—C29_2	120.67 (13)
C31_1—C30_1—H30_1	119.5	C31_2—C30_2—H30_2	119.7
C29_1—C30_1—H30_1	119.5	C29_2—C30_2—H30_2	119.7
C30_1—C31_1—C32_1	120.01 (14)	C30_2—C31_2—C32_2	120.20 (14)
C30_1—C31_1—H31_1	120.0	C30_2—C31_2—H31_2	119.9
C32_1—C31_1—H31_1	120.0	C32_2—C31_2—H31_2	119.9
C33_1—C32_1—C31_1	119.56 (14)	C33_2—C32_2—C31_2	119.61 (13)
C33_1—C32_1—H32_1	120.2	C33_2—C32_2—H32_2	120.2
C31_1—C32_1—H32_1	120.2	C31_2—C32_2—H32_2	120.2
C32_1—C33_1—C34_1	120.55 (14)	C32_2—C33_2—C34_2	120.32 (13)
C32_1—C33_1—H33_1	119.7	C32_2—C33_2—H33_2	119.8
C34_1—C33_1—H33_1	119.7	C34_2—C33_2—H33_2	119.8
C33_1—C34_1—C29_1	120.32 (13)	C33_2—C34_2—C29_2	120.55 (13)
C33_1—C34_1—H34_1	119.8	C33_2—C34_2—H34_2	119.7
C29_1—C34_1—H34_1	119.8	C29_2—C34_2—H34_2	119.7
C40_1—C35_1—C36_1	119.26 (15)	C40_2—C35_2—C36_2	119.25 (14)
C40_1—C35_1—C14_1	122.55 (15)	C40_2—C35_2—C14_2	121.69 (13)
C36_1—C35_1—C14_1	118.15 (13)	C36_2—C35_2—C14_2	119.06 (13)
C35_1—C36_1—C37_1	120.20 (18)	C37_2—C36_2—C35_2	120.23 (17)
C35_1—C36_1—H36_1	119.9	C37_2—C36_2—H36_2	119.9
C37_1—C36_1—H36_1	119.9	C35_2—C36_2—H36_2	119.9
C38_1—C37_1—C36_1	120.1 (2)	C38_2—C37_2—C36_2	120.17 (19)
C38_1—C37_1—H37_1	119.9	C38_2—C37_2—H37_2	119.9
C36_1—C37_1—H37_1	119.9	C36_2—C37_2—H37_2	119.9
C39_1—C38_1—C37_1	119.99 (18)	C39_2—C38_2—C37_2	119.96 (17)
C39_1—C38_1—H38_1	120.0	C39_2—C38_2—H38_2	120.0
C37_1—C38_1—H38_1	120.0	C37_2—C38_2—H38_2	120.0
C38_1—C39_1—C40_1	120.4 (2)	C38_2—C39_2—C40_2	120.44 (19)
C38_1—C39_1—H39_1	119.8	C38_2—C39_2—H39_2	119.8
C40_1—C39_1—H39_1	119.8	C40_2—C39_2—H39_2	119.8
C35_1—C40_1—C39_1	119.9 (2)	C35_2—C40_2—C39_2	119.95 (18)
C35_1—C40_1—H40_1	120.0	C35_2—C40_2—H40_2	120.0
C39_1—C40_1—H40_1	120.0	C39_2—C40_2—H40_2	120.0
C42_1—C41_1—C46_1	119.32 (12)	C42_2—C41_2—C46_2	119.39 (11)
C42_1—C41_1—C19_1	119.83 (11)	C42_2—C41_2—C19_2	119.00 (11)
C46_1—C41_1—C19_1	120.77 (11)	C46_2—C41_2—C19_2	121.38 (11)
C43_1—C42_1—C41_1	119.96 (12)	C41_2—C42_2—C43_2	120.33 (12)
C43_1—C42_1—H42_1	120.0	C41_2—C42_2—H42_2	119.8
C41_1—C42_1—H42_1	120.0	C43_2—C42_2—H42_2	119.8
C44_1—C43_1—C42_1	120.47 (13)	C44_2—C43_2—C42_2	120.04 (12)
C44_1—C43_1—H43_1	119.8	C44_2—C43_2—H43_2	120.0
C42_1—C43_1—H43_1	119.8	C42_2—C43_2—H43_2	120.0
C43_1—C44_1—C45_1	119.84 (13)	C43_2—C44_2—C45_2	120.04 (12)
C43_1—C44_1—H44_1	120.1	C43_2—C44_2—H44_2	120.0
C45_1—C44_1—H44_1	120.1	C45_2—C44_2—H44_2	120.0
C46_1—C45_1—C44_1	119.99 (12)	C44_2—C45_2—C46_2	120.23 (12)
C46_1—C45_1—H45_1	120.0	C44_2—C45_2—H45_2	119.9
C44_1—C45_1—H45_1	120.0	C46_2—C45_2—H45_2	119.9
C45_1—C46_1—C41_1	120.39 (12)	C45_2—C46_2—C41_2	119.97 (12)
C45_1—C46_1—H46_1	119.8	C45_2—C46_2—H46_2	120.0
C41_1—C46_1—H46_1	119.8	C41_2—C46_2—H46_2	120.0
			
N2_1—Ni1_1—N1_1—C3_1	39.81 (10)	C42_1—C41_1—C46_1—C45_1	−1.5 (2)
N4_1—Ni1_1—N1_1—C3_1	−136.86 (10)	C19_1—C41_1—C46_1—C45_1	−178.15 (12)
N2_1—Ni1_1—N1_1—C20_1	−143.06 (10)	N3_2—Ni1_2—N2_2—C5_2	154.78 (11)
N4_1—Ni1_1—N1_1—C20_1	40.28 (10)	N1_2—Ni1_2—N2_2—C5_2	−26.71 (11)
N1_1—Ni1_1—N2_1—C5_1	−19.48 (10)	N3_2—Ni1_2—N2_2—C8_2	−20.15 (11)
N3_1—Ni1_1—N2_1—C5_1	160.83 (10)	N1_2—Ni1_2—N2_2—C8_2	158.36 (11)
N1_1—Ni1_1—N2_1—C8_1	157.04 (11)	N3_2—Ni1_2—N4_2—C18_2	165.64 (10)
N3_1—Ni1_1—N2_1—C8_1	−22.64 (11)	N1_2—Ni1_2—N4_2—C18_2	−12.89 (11)
N1_1—Ni1_1—N4_1—C18_1	−14.98 (11)	N3_2—Ni1_2—N4_2—C15_2	−27.18 (10)
N3_1—Ni1_1—N4_1—C18_1	164.71 (11)	N1_2—Ni1_2—N4_2—C15_2	154.29 (10)
N1_1—Ni1_1—N4_1—C15_1	154.90 (11)	C20_2—N1_2—C3_2—C4_2	154.90 (12)
N3_1—Ni1_1—N4_1—C15_1	−25.42 (11)	Ni1_2—N1_2—C3_2—C4_2	−25.72 (17)
C20_1—N1_1—C3_1—C4_1	144.70 (12)	C20_2—N1_2—C3_2—C2_2	−31.32 (15)
Ni1_1—N1_1—C3_1—C4_1	−37.95 (16)	Ni1_2—N1_2—C3_2—C2_2	148.07 (9)
C20_1—N1_1—C3_1—C2_1	−46.59 (15)	O1_2—C2_2—C3_2—N1_2	−37.75 (17)
Ni1_1—N1_1—C3_1—C2_1	130.76 (10)	C1_2—C2_2—C3_2—N1_2	143.45 (13)
O1_1—C2_1—C3_1—N1_1	39.90 (19)	O1_2—C2_2—C3_2—C4_2	136.28 (13)
C1_1—C2_1—C3_1—N1_1	−130.33 (13)	C1_2—C2_2—C3_2—C4_2	−42.52 (18)
O1_1—C2_1—C3_1—C4_1	−151.09 (14)	N1_2—C3_2—C4_2—C5_2	−7.22 (19)
C1_1—C2_1—C3_1—C4_1	38.67 (17)	C2_2—C3_2—C4_2—C5_2	179.12 (11)
N1_1—C3_1—C4_1—C5_1	2.82 (19)	N1_2—C3_2—C4_2—C23_2	169.86 (12)
C2_1—C3_1—C4_1—C5_1	−165.34 (12)	C2_2—C3_2—C4_2—C23_2	−3.80 (18)
N1_1—C3_1—C4_1—C23_1	−170.12 (11)	C8_2—N2_2—C5_2—C4_2	−178.62 (12)
C2_1—C3_1—C4_1—C23_1	21.71 (18)	Ni1_2—N2_2—C5_2—C4_2	5.57 (18)
C8_1—N2_1—C5_1—C4_1	177.17 (12)	C8_2—N2_2—C5_2—C6_2	0.42 (14)
Ni1_1—N2_1—C5_1—C4_1	−5.69 (18)	Ni1_2—N2_2—C5_2—C6_2	−175.40 (9)
C8_1—N2_1—C5_1—C6_1	−1.73 (14)	C3_2—C4_2—C5_2—N2_2	18.0 (2)
Ni1_1—N2_1—C5_1—C6_1	175.41 (8)	C23_2—C4_2—C5_2—N2_2	−159.19 (12)
C3_1—C4_1—C5_1—N2_1	20.07 (19)	C3_2—C4_2—C5_2—C6_2	−160.86 (13)
C23_1—C4_1—C5_1—N2_1	−166.94 (11)	C23_2—C4_2—C5_2—C6_2	21.93 (19)
C3_1—C4_1—C5_1—C6_1	−161.20 (12)	N2_2—C5_2—C6_2—C7_2	−2.88 (16)
C23_1—C4_1—C5_1—C6_1	11.78 (19)	C4_2—C5_2—C6_2—C7_2	176.14 (13)
N2_1—C5_1—C6_1—C7_1	−0.22 (15)	C5_2—C6_2—C7_2—C8_2	4.00 (15)
C4_1—C5_1—C6_1—C7_1	−179.09 (12)	C5_2—N2_2—C8_2—C9_2	−166.97 (12)
C5_1—C6_1—C7_1—C8_1	2.00 (15)	Ni1_2—N2_2—C8_2—C9_2	8.74 (18)
C5_1—N2_1—C8_1—C9_1	−167.75 (12)	C5_2—N2_2—C8_2—C7_2	2.07 (14)
Ni1_1—N2_1—C8_1—C9_1	15.17 (18)	Ni1_2—N2_2—C8_2—C7_2	177.78 (9)
C5_1—N2_1—C8_1—C7_1	2.96 (14)	C6_2—C7_2—C8_2—C9_2	165.08 (13)
Ni1_1—N2_1—C8_1—C7_1	−174.12 (9)	C6_2—C7_2—C8_2—N2_2	−3.91 (15)
C6_1—C7_1—C8_1—N2_1	−3.17 (15)	N2_2—C8_2—C9_2—C10_2	8.94 (19)
C6_1—C7_1—C8_1—C9_1	167.39 (13)	C7_2—C8_2—C9_2—C10_2	−158.39 (13)
N2_1—C8_1—C9_1—C10_1	4.21 (19)	N2_2—C8_2—C9_2—C29_2	−176.68 (11)
C7_1—C8_1—C9_1—C10_1	−164.99 (12)	C7_2—C8_2—C9_2—C29_2	15.99 (19)
N2_1—C8_1—C9_1—C29_1	179.48 (11)	C13_2—N3_2—C10_2—C9_2	169.02 (12)
C7_1—C8_1—C9_1—C29_1	10.28 (19)	Ni1_2—N3_2—C10_2—C9_2	−13.63 (17)
C13_1—N3_1—C10_1—C9_1	171.29 (12)	C13_2—N3_2—C10_2—C11_2	−3.32 (14)
Ni1_1—N3_1—C10_1—C9_1	−8.88 (18)	Ni1_2—N3_2—C10_2—C11_2	174.03 (9)
C13_1—N3_1—C10_1—C11_1	−2.88 (14)	C8_2—C9_2—C10_2—N3_2	−6.32 (19)
Ni1_1—N3_1—C10_1—C11_1	176.95 (9)	C29_2—C9_2—C10_2—N3_2	179.42 (11)
C8_1—C9_1—C10_1—N3_1	−7.29 (19)	C8_2—C9_2—C10_2—C11_2	164.83 (12)
C29_1—C9_1—C10_1—N3_1	177.58 (11)	C29_2—C9_2—C10_2—C11_2	−9.42 (19)
C8_1—C9_1—C10_1—C11_1	165.98 (12)	N3_2—C10_2—C11_2—C12_2	4.29 (15)
C29_1—C9_1—C10_1—C11_1	−9.14 (19)	C9_2—C10_2—C11_2—C12_2	−167.93 (12)
N3_1—C10_1—C11_1—C12_1	4.04 (15)	C10_2—C11_2—C12_2—C13_2	−3.36 (15)
C9_1—C10_1—C11_1—C12_1	−170.06 (13)	C10_2—N3_2—C13_2—C14_2	178.23 (12)
C10_1—C11_1—C12_1—C13_1	−3.42 (15)	Ni1_2—N3_2—C13_2—C14_2	0.87 (18)
C10_1—N3_1—C13_1—C14_1	178.53 (12)	C10_2—N3_2—C13_2—C12_2	1.21 (14)
Ni1_1—N3_1—C13_1—C14_1	−1.30 (19)	Ni1_2—N3_2—C13_2—C12_2	−176.14 (9)
C10_1—N3_1—C13_1—C12_1	0.73 (14)	C11_2—C12_2—C13_2—N3_2	1.43 (16)
Ni1_1—N3_1—C13_1—C12_1	−179.10 (9)	C11_2—C12_2—C13_2—C14_2	−175.58 (13)
C11_1—C12_1—C13_1—N3_1	1.77 (16)	N3_2—C13_2—C14_2—C15_2	−12.8 (2)
C11_1—C12_1—C13_1—C14_1	−176.01 (13)	C12_2—C13_2—C14_2—C15_2	163.74 (13)
N3_1—C13_1—C14_1—C15_1	−12.0 (2)	N3_2—C13_2—C14_2—C35_2	169.71 (12)
C12_1—C13_1—C14_1—C15_1	165.47 (13)	C12_2—C13_2—C14_2—C35_2	−13.7 (2)
N3_1—C13_1—C14_1—C35_1	168.68 (12)	C13_2—C14_2—C15_2—N4_2	0.21 (19)
C12_1—C13_1—C14_1—C35_1	−13.9 (2)	C35_2—C14_2—C15_2—N4_2	177.64 (12)
C18_1—N4_1—C15_1—C14_1	−167.68 (12)	C13_2—C14_2—C15_2—C16_2	−169.01 (12)
Ni1_1—N4_1—C15_1—C14_1	20.71 (18)	C35_2—C14_2—C15_2—C16_2	8.4 (2)
C18_1—N4_1—C15_1—C16_1	2.93 (14)	C18_2—N4_2—C15_2—C14_2	−166.51 (12)
Ni1_1—N4_1—C15_1—C16_1	−168.68 (9)	Ni1_2—N4_2—C15_2—C14_2	24.16 (17)
C13_1—C14_1—C15_1—N4_1	2.1 (2)	C18_2—N4_2—C15_2—C16_2	4.26 (13)
C35_1—C14_1—C15_1—N4_1	−178.56 (12)	Ni1_2—N4_2—C15_2—C16_2	−165.08 (8)
C13_1—C14_1—C15_1—C16_1	−166.96 (13)	C14_2—C15_2—C16_2—C17_2	166.20 (12)
C35_1—C14_1—C15_1—C16_1	12.4 (2)	N4_2—C15_2—C16_2—C17_2	−4.26 (14)
N4_1—C15_1—C16_1—C17_1	−3.01 (16)	C15_2—C16_2—C17_2—C18_2	2.45 (14)
C14_1—C15_1—C16_1—C17_1	167.33 (13)	C15_2—N4_2—C18_2—C19_2	175.66 (11)
C15_1—C16_1—C17_1—C18_1	1.81 (16)	Ni1_2—N4_2—C18_2—C19_2	−14.99 (17)
C15_1—N4_1—C18_1—C19_1	176.72 (12)	C15_2—N4_2—C18_2—C17_2	−2.73 (13)
Ni1_1—N4_1—C18_1—C19_1	−11.76 (18)	Ni1_2—N4_2—C18_2—C17_2	166.61 (8)
C15_1—N4_1—C18_1—C17_1	−1.80 (14)	C16_2—C17_2—C18_2—N4_2	0.13 (14)
Ni1_1—N4_1—C18_1—C17_1	169.73 (9)	C16_2—C17_2—C18_2—C19_2	−178.21 (12)
C16_1—C17_1—C18_1—N4_1	−0.04 (15)	N4_2—C18_2—C19_2—C20_2	22.87 (18)
C16_1—C17_1—C18_1—C19_1	−178.51 (13)	C17_2—C18_2—C19_2—C20_2	−158.98 (12)
N4_1—C18_1—C19_1—C20_1	21.94 (19)	N4_2—C18_2—C19_2—C41_2	−166.25 (11)
C17_1—C18_1—C19_1—C20_1	−159.78 (13)	C17_2—C18_2—C19_2—C41_2	11.90 (18)
N4_1—C18_1—C19_1—C41_1	−165.57 (11)	C18_2—C19_2—C20_2—N1_2	7.98 (18)
C17_1—C18_1—C19_1—C41_1	12.70 (19)	C41_2—C19_2—C20_2—N1_2	−162.37 (11)
C18_1—C19_1—C20_1—N1_1	5.43 (18)	C18_2—C19_2—C20_2—C21_2	−168.41 (11)
C41_1—C19_1—C20_1—N1_1	−166.64 (11)	C41_2—C19_2—C20_2—C21_2	21.23 (18)
C18_1—C19_1—C20_1—C21_1	−167.78 (12)	C3_2—N1_2—C20_2—C19_2	135.71 (12)
C41_1—C19_1—C20_1—C21_1	20.15 (19)	Ni1_2—N1_2—C20_2—C19_2	−43.69 (15)
C3_1—N1_1—C20_1—C19_1	135.50 (12)	C3_2—N1_2—C20_2—C21_2	−47.71 (15)
Ni1_1—N1_1—C20_1—C19_1	−41.81 (16)	Ni1_2—N1_2—C20_2—C21_2	132.89 (9)
C3_1—N1_1—C20_1—C21_1	−50.75 (15)	C19_2—C20_2—C21_2—O2_2	−156.63 (13)
Ni1_1—N1_1—C20_1—C21_1	131.93 (10)	N1_2—C20_2—C21_2—O2_2	26.78 (18)
C19_1—C20_1—C21_1—O2_1	−160.19 (13)	C19_2—C20_2—C21_2—C22_2	30.58 (18)
N1_1—C20_1—C21_1—O2_1	26.20 (18)	N1_2—C20_2—C21_2—C22_2	−146.01 (12)
C19_1—C20_1—C21_1—C22_1	27.72 (19)	C3_2—C4_2—C23_2—C28_2	−66.61 (18)
N1_1—C20_1—C21_1—C22_1	−145.89 (12)	C5_2—C4_2—C23_2—C28_2	110.57 (15)
C3_1—C4_1—C23_1—C28_1	47.55 (18)	C3_2—C4_2—C23_2—C24_2	115.13 (15)
C5_1—C4_1—C23_1—C28_1	−125.51 (14)	C5_2—C4_2—C23_2—C24_2	−67.69 (17)
C3_1—C4_1—C23_1—C24_1	−130.42 (14)	C28_2—C23_2—C24_2—C25_2	−0.6 (2)
C5_1—C4_1—C23_1—C24_1	56.53 (17)	C4_2—C23_2—C24_2—C25_2	177.65 (14)
C28_1—C23_1—C24_1—C25_1	−2.4 (2)	C23_2—C24_2—C25_2—C26_2	0.2 (3)
C4_1—C23_1—C24_1—C25_1	175.60 (13)	C24_2—C25_2—C26_2—C27_2	0.6 (3)
C23_1—C24_1—C25_1—C26_1	1.1 (2)	C25_2—C26_2—C27_2—C28_2	−0.8 (3)
C24_1—C25_1—C26_1—C27_1	1.1 (2)	C26_2—C27_2—C28_2—C23_2	0.4 (3)
C25_1—C26_1—C27_1—C28_1	−2.0 (2)	C24_2—C23_2—C28_2—C27_2	0.4 (2)
C26_1—C27_1—C28_1—C23_1	0.7 (2)	C4_2—C23_2—C28_2—C27_2	−177.98 (15)
C24_1—C23_1—C28_1—C27_1	1.4 (2)	C8_2—C9_2—C29_2—C34_2	−119.00 (14)
C4_1—C23_1—C28_1—C27_1	−176.57 (13)	C10_2—C9_2—C29_2—C34_2	55.43 (17)
C8_1—C9_1—C29_1—C30_1	59.97 (17)	C8_2—C9_2—C29_2—C30_2	59.66 (17)
C10_1—C9_1—C29_1—C30_1	−124.73 (14)	C10_2—C9_2—C29_2—C30_2	−125.91 (14)
C8_1—C9_1—C29_1—C34_1	−116.99 (14)	C34_2—C29_2—C30_2—C31_2	−1.3 (2)
C10_1—C9_1—C29_1—C34_1	58.30 (18)	C9_2—C29_2—C30_2—C31_2	−179.96 (14)
C34_1—C29_1—C30_1—C31_1	0.6 (2)	C29_2—C30_2—C31_2—C32_2	0.3 (3)
C9_1—C29_1—C30_1—C31_1	−176.44 (13)	C30_2—C31_2—C32_2—C33_2	0.8 (3)
C29_1—C30_1—C31_1—C32_1	−0.2 (2)	C31_2—C32_2—C33_2—C34_2	−1.0 (2)
C30_1—C31_1—C32_1—C33_1	−0.4 (2)	C32_2—C33_2—C34_2—C29_2	0.1 (2)
C31_1—C32_1—C33_1—C34_1	0.6 (2)	C30_2—C29_2—C34_2—C33_2	1.0 (2)
C32_1—C33_1—C34_1—C29_1	−0.2 (2)	C9_2—C29_2—C34_2—C33_2	179.70 (12)
C30_1—C29_1—C34_1—C33_1	−0.4 (2)	C15_2—C14_2—C35_2—C40_2	−68.11 (19)
C9_1—C29_1—C34_1—C33_1	176.56 (13)	C13_2—C14_2—C35_2—C40_2	109.40 (17)
C15_1—C14_1—C35_1—C40_1	−97.1 (2)	C15_2—C14_2—C35_2—C36_2	111.32 (16)
C13_1—C14_1—C35_1—C40_1	82.3 (2)	C13_2—C14_2—C35_2—C36_2	−71.18 (18)
C15_1—C14_1—C35_1—C36_1	85.30 (18)	C40_2—C35_2—C36_2—C37_2	−0.2 (3)
C13_1—C14_1—C35_1—C36_1	−95.35 (17)	C14_2—C35_2—C36_2—C37_2	−179.63 (16)
C40_1—C35_1—C36_1—C37_1	0.1 (3)	C35_2—C36_2—C37_2—C38_2	−0.3 (3)
C14_1—C35_1—C36_1—C37_1	177.75 (17)	C36_2—C37_2—C38_2—C39_2	0.6 (3)
C35_1—C36_1—C37_1—C38_1	1.3 (3)	C37_2—C38_2—C39_2—C40_2	−0.4 (4)
C36_1—C37_1—C38_1—C39_1	−1.3 (4)	C36_2—C35_2—C40_2—C39_2	0.4 (3)
C37_1—C38_1—C39_1—C40_1	0.0 (4)	C14_2—C35_2—C40_2—C39_2	179.78 (18)
C36_1—C35_1—C40_1—C39_1	−1.4 (3)	C38_2—C39_2—C40_2—C35_2	−0.1 (3)
C14_1—C35_1—C40_1—C39_1	−179.0 (2)	C20_2—C19_2—C41_2—C42_2	56.12 (17)
C38_1—C39_1—C40_1—C35_1	1.4 (4)	C18_2—C19_2—C41_2—C42_2	−114.65 (14)
C20_1—C19_1—C41_1—C42_1	57.29 (18)	C20_2—C19_2—C41_2—C46_2	−129.46 (14)
C18_1—C19_1—C41_1—C42_1	−115.05 (14)	C18_2—C19_2—C41_2—C46_2	59.77 (16)
C20_1—C19_1—C41_1—C46_1	−126.06 (14)	C46_2—C41_2—C42_2—C43_2	−0.2 (2)
C18_1—C19_1—C41_1—C46_1	61.60 (16)	C19_2—C41_2—C42_2—C43_2	174.36 (13)
C46_1—C41_1—C42_1—C43_1	0.9 (2)	C41_2—C42_2—C43_2—C44_2	0.7 (2)
C19_1—C41_1—C42_1—C43_1	177.64 (13)	C42_2—C43_2—C44_2—C45_2	−0.3 (2)
C41_1—C42_1—C43_1—C44_1	0.6 (2)	C43_2—C44_2—C45_2—C46_2	−0.7 (2)
C42_1—C43_1—C44_1—C45_1	−1.6 (2)	C44_2—C45_2—C46_2—C41_2	1.3 (2)
C43_1—C44_1—C45_1—C46_1	1.0 (2)	C42_2—C41_2—C46_2—C45_2	−0.8 (2)
C44_1—C45_1—C46_1—C41_1	0.5 (2)	C19_2—C41_2—C46_2—C45_2	−175.22 (12)

### Hydrogen-bond geometry (Å, º)

**Table:**

*D*—H···*A*	*D*—H	H···*A*	*D*···*A*	*D*—H···*A*
C28_1—H28_1···O2_1	0.95	2.60	3.4517 (17)	149
C42_1—H42_1···O1_1	0.95	2.55	3.3855 (17)	147
C42_2—H42_2···O1_2	0.95	2.66	3.5120 (17)	149
C6_1—H6_1···O2_1^i^	0.95	2.49	3.3714 (16)	155
C6_2—H6_2···O2_2^ii^	0.95	2.57	3.3569 (17)	140
C17_2—H17_2···O1_2^iii^	0.95	2.40	3.2977 (16)	158

Symmetry codes: (i) −*x*+1, −*y*+1, −*z*+2; (ii) −*x*+1, −*y*+2, −*z*+1; (iii) −*x*+1, −*y*+1, −*z*+1.
